# Pleiotropic roles of LAMMER kinase, Lkh1 in stress responses and virulence of *Cryptococcus neoformans*

**DOI:** 10.3389/fcimb.2024.1369301

**Published:** 2024-05-07

**Authors:** Sunhak Kwon, Yeseul Choi, Eui-Seong Kim, Kyung-Tae Lee, Yong-Sun Bahn, Kwang-Woo Jung

**Affiliations:** ^1^ Advanced Radiation Technology Institute, Korea Atomic Energy Research Institute, Jeongeup, Jeonbuk, Republic of Korea; ^2^ Department of Biotechnology, College of Life Science and Biotechnology, Yonsei University, Seoul, Republic of Korea; ^3^ Korea Zoonosis Research Institute, Jeonbuk National University, Iksan, Jeonbuk, Republic of Korea

**Keywords:** LAMMER kinase, *Cryptococcus*, stress response, virulence, antifungal drug resistance

## Abstract

Dual-specificity LAMMER kinases are highly evolutionarily conserved in eukaryotes and play pivotal roles in diverse physiological processes, such as growth, differentiation, and stress responses. Although the functions of LAMMER kinase in fungal pathogens in pathogenicity and stress responses have been characterized, its role in *Cryptococcus neoformans*, a human fungal pathogen and a model yeast of basidiomycetes, remains elusive. In this study, we identified a *LKH1* homologous gene and constructed a strain with a deleted *LKH1* and a complemented strain. Similar to other fungi, the *lkh1*Δ mutant showed intrinsic growth defects. We observed that *C. neoformans* Lkh1 was involved in diverse stress responses, including oxidative stress and cell wall stress. Particularly, Lkh1 regulates DNA damage responses in Rad53-dependent and -independent manners. Furthermore, the absence of *LKH1* reduced basidiospore formation. Our observations indicate that Lkh1 becomes hyperphosphorylated upon treatment with rapamycin, a TOR protein inhibitor. Notably, *LKH1* deletion led to defects in melanin synthesis and capsule formation. Furthermore, we found that the deletion of *LKH1* led to the avirulence of *C. neoformans* in a systemic cryptococcosis murine model. Taken together, Lkh1 is required for the stress response, sexual differentiation, and virulence of *C. neoformans*.

## 1 Introduction

In cell signaling cascades, protein phosphorylation and dephosphorylation serve as pivotal switches, orchestrating the activation or deactivation of signaling cascades by facilitating alterations in protein conformation, protein-protein interactions, and subcellular localization. These dynamic processes play critical roles in regulating various biological phenomena. Most protein phosphorylation occurs at serine, threonine, and tyrosine residues. Occasionally, histidine and aspartate residues are phosphorylated; however, these modifications are less prevalent and exhibit lower stability than those of serine, threonine, and tyrosine (Duclos et al., 1991). Given the importance of protein phosphorylation in cell signaling cascades, protein kinases are evolutionarily conserved across species (Manning et al., 2002).

Within the evolutionarily conserved kinases, LAMMER kinase stands out, characterized by the presence of a conserved motif, ‘EHLAMMERILG’ located in the subdomain X of the kinase catalytic domain (Yun et al., 1994). This kinase plays crucial roles in various physiological processes, such as differentiation, growth, and stress responses, from yeasts to humans. The LAMMER kinase is a dual-specific kinase that exhibits autophosphorylation activity in *Drosophila*, humans, and *Saccharomyces cerevisiae* (Lee et al., 1996). In humans, three LAMMER kinase isoforms, CLK1 (Cdc-like kinase 1), CLK2, and CLK3, are located at different chromosomal loci (Hanes et al., 1994; Talmadge et al., 1998). hCLKs play important roles in diverse cellular responses, including neuronal differentiation, cell cycle progression, and apoptosis (Myers et al., 1994; Jiang et al., 2003). Similar to humans, *Arabidopsis thaliana* possesses three LAMMER kinase homologs: AFC1, AFC2, and AFC3 (Savaldi-Goldstein et al., 2003). AFC1, but not AFC2, restores Ste12-dependent pseudohyphal growth in *S. cerevisiae* (Bender and Fink, 1994). In contrast to higher eukaryotes, which harbor several LAMMER kinase variants, the model yeasts *S. cerevisiae* and *Schizosaccharomyces pombe* have a singular LAMMER kinase. Unlike other human and plant LAMMER kinases, the deletion of *KNS1*, which encodes a LAMMER kinase in *S. cerevisiae*, does not affect the viability, growth, and sporulation efficiency (Padmanabha et al., 1991). Although the *kns1*Δ mutant in the S288c background does not exhibit noticeable phenotypes compared to the wild-type (WT), its regulatory mechanism has been elucidated. Kns1 undergoes autophosphorylation and its localization becomes enriched in the nucleus under TOR-suppressed conditions (Lee et al., 2012). Furthermore, Kns1 is involved in ribosome and tRNA synthesis through RNA polymerase III subunit Rpc53 phosphorylation and the regulatory subunit of the CK2 protein kinase, Ckb1 (Lee et al., 2012; Sanchez-Casalongue et al., 2015). The deletion of *LKH1*, which encodes a LAMMER kinase in *S. pombe*, results in increased susceptibility to oxidative stress by reducing the expression levels of genes encoding catalase and superoxide dismutase (Park et al., 2003). Furthermore, SpLkh1 acts as a negative filamentous growth regulator and is involved in cell wall biosynthesis (Park et al., 2003; Cho et al., 2010).

Besides the model budding and fission yeasts, the roles of LAMMER kinase and its functional regulatory mechanisms in fungal pathogens have been elucidated. In *Candida albicans*, *CaKNS1* is involved in hyphal morphogenesis and stress responses such as cell wall stress and DNA replicative stress (Lim et al., 2020). Notably, *CaKNS1* is required for the full virulence of *C. albicans* (Park and Park, 2011). In *Aspergillus fumigatus*, *LkhA* deletion results in growth and sexual differentiation defects. Similar to the *Cakns1*Δ mutant, the Δ*lhkA* mutant exhibits attenuated virulence in the zebrafish larvae model (Lim et al., 2021). In the plant fungal pathogen *Magnaporthe oryzae*, the *kns1*Δ mutant exhibits decreased growth and conidiation, similar to other filamentous fungi. Furthermore, the virulence of the Δ*kns1* mutant is significantly reduced in a rice infection model (Li et al., 2022). In *Ustilago maydis*, UnLhk1 is required for DNA damage, meiotic recombination, and chromosomal segregation. However, the pathogenicity of the *Unlkh1*Δ mutant has not yet been determined (de Sena-Tomás et al., 2015).

*Cryptococcus neoformans* is a human fungal pathogen belonging to the basidiomycetes and is particularly prevalent in immunocompromised patients. Without appropriate therapeutic treatment, the mortality rate increases significantly (Idnurm et al., 2005). Briefly, spores or desiccated *C. neoformans* from the environment are inhaled into the lungs via the respiratory tract (Ellis and Pfeiffer, 1990; Velagapudi et al., 2009). Next, the *C. neoformans* disseminates to other organs in the host and subsequently migrates to the central nervous system, leading to life-threatening meningitis (Gottfredsson and Perfect, 2000; Kwon-Chung et al., 2000). A recent study estimates that 152,000 cases of cryptococcal meningitis occurred in patients with HIV/AIDS, resulting in nearly 112,000 deaths in 2020 (Rajasingham et al., 2022). Furthermore, the incidence of cryptococcal infections has increased in patients with COVID-19 (Regalla et al., 2022). *C. neoformans* serves as an attractive fungal model for studying host-pathogen interactions (Perfect, 2014).

Although the role of LAMMER kinase Lkh1 in growth, stress response, and virulence has been clarified in ascomycetous human fungal pathogens, the pathobiological functions of the Lkh1 homolog in *C. neoformans* remain elusive. To explore the roles of Lkh1, we identified the *LKH1* homologous gene and performed phenotypic analyses using *lkh1*Δ mutants. Our findings reveal that the deletion of *LKH1* led to defects in growth, sexual differentiation, and stress responses. Particularly, Lkh1 was required for virulence in the mice model. Therefore, we propose that Lkh1 is vital for pathogenicity, stress response, and differentiation of the human fungal pathogen *Cryptococcus*.

## 2 Materials and methods

### 2.1 Ethics statement

Animal care and research were approved after deliberation by the Institutional Animal Care and Use Committee of the Experimental Animal Center at Jeonbuk National University (Approval number: JBNU 2022-092). All experiments were conducted per experimental ethics guidelines. The animal experiments were conducted at the Core Facility Center for Zoonosis Research (Jeonbuk National University, South Korea).

### 2.2 Strains and growth condition

The *C. neoformans* strains used in the experiments were listed in **Supplementary Table S1** and were cultured in yeast extract-peptone-dextrose (YPD) medium. The V8 medium used for the mating assay contained 5% V8 juice, 0.5 g/L of KH_2_PO_4_, and 4% bacto-agar with the pH adjusted to 5 using a KOH solution (Jung et al., 2018). For the melanin production assay, equivalent numbers of cells were spotted on a dopamine agar medium with varying glucose concentrations (0%, 0.1%, or 0.2%) (Lee et al., 2019). For the capsule assay, each strain was incubated in Littman’s medium (Littman, 1958) and capsule thickness was measured as previously described (Jang et al., 2022). To assess the growth of strains using a single amino acid as the sole nitrogen source, equivalent amounts of cells were subcultured in a minimal medium (YNB without amino acids and ammonium sulfate) containing specific nitrogen sources (2 mM proline, 2 mM isoleucine, and 2 mM lysine).

### 2.3 Construction of mutant strains

Gene disruption information was obtained from FungiDB (www.fungiDB.org). *LKH1* (CNAG_00683) was disrupted by double-joint PCR using split markers and Cryptococcus transformation as described previously (Fan and Lin, 2018; Jung et al., 2018). **Supplementary Table S2** describes the primers for generating the 5’- and 3’-flanking regions of *LKH1* and the nourseothricin resistance (*NAT*) and G418 resistance (*NEO*) dominant selectable markers. Gel-extracted deletion cassettes, the SgRNA gene, and Cas9 were transformed using a BioRad gene pulser (Cat# 1652086). The *rad53*Δ *lkh1*Δ double mutant and *mpk1*Δ *lkh1*Δ double mutant were constructed by introducing the *lkh1*Δ::*NEO* allele into the *rad53*Δ mutant (YSB3785) and *mpk1*Δ mutant (YSB3814) through the Cryptococcus transformation. The Rad53-4×FLAG *lkh1*Δ strain was constructed by introducing the *lkh1*Δ::*NAT* allele into the Rad53-4×FLAG mutant (YSB3806) using the Cryptococcus transformation. To produce Lkh1-4×FLAG strains, three PCR product fragments were amplified. The 3’-exon region of *LKH1* was amplified using primers J1966 and J1967 and H99 genomic DNA as the template. The 4×FLAG-HOG1ter-NEO fragment was amplified using primers B6567 and B354 and the plasmid pNEO-4×FLAG as the template (So et al., 2017). The 3’-flanking region of the *LKH1* was amplified using primers J1969 and J1968 and H99 genomic DNA as the template. Next, a fusion fragment, including the 3’-exon region of *LKH1* and 5’-split region of the 4×FLAG-HOG1ter-NEO fragment, was amplified using primers B1886 and J1966 and the mixed first-round PCR product as the template. A fusion fragment containing the 3’-split region of the 4×FLAG-HOG1ter-NEO fragment and the 3’-flanking region of the *LKH1* gene was amplified using primers J1969 and B1887 and the combined first-round PCR products as a template. The two DJ-PCR products were mixed and introduced into the H99 strain via biolistic transformation (Jung et al., 2018). The Lkh1-4×FLAG *sit4*Δ strain or the Lkh1-4×FLAG *sch9*Δ strain were constructed by introducing the *sit4*Δ::*NAT* allele into the Lkh1-4×FLAG mutant (KW1755) using the Cryptococcus transformation or introducing *sch9*Δ::*NAT* allele into the Lkh1-4×FLAG mutant (KW1755) using the biolistic transformation. To confirm the gene disruption, Southern blot analysis was performed using a DIG-labeled gene-specific probe (**Supplementary Figure S1**).

### 2.4 Construction of *LKH1* and *LKH1*-KD (kinase-dead) complemented strains

To validate the observed phenotypes in the *lkh1*Δ mutant, we constructed the corresponding complemented strains as follows. The *LKH1* gene fragment containing the promoter, open reading frame (ORF), and the terminator was divided into two fragments and amplified by PCR using the primer pairs J718/J723 and J1711/J1712 for the *LKH1-L* and *LKH1-R* parts, respectively. Each amplified gene fragment was cloned into the plasmid pJET 2.1 to produce pJET-*LKH1-L* (KWE174) and pJET-*LKH1-R* (KWE176). After confirming the DNA sequence of each part of *LKH1*, the BamHI restriction enzyme-digested pJET-*LKH1-R* insert was subcloned into pJET-*LKH1-L* to generate the plasmid pJET-*LKH1-LR* (KWE181), which contains a genomic fragment of the *LKH1* gene. Next, the XhoI restriction enzyme-digested pJET-*LKH1-LR* insert was subcloned into plasmid pTOP-NEO containing the NEO-selection marker to produce pNEO-*LKH1* (KWE188). The linearized pNEO-*LKH1*, digested by SnaBI, was introduced into the native *LKH1* locus of the *lkh1*Δ mutant (KW1451) by biolistic transformation. To confirm the correct *LKH1* gene insertion, we conducted diagnostic PCR using primers J1895 and J1705. To construct *LKH1-KD* strains, we generated *LKH1* kinase-dead allele (D546A; *LKH1KD*) as follows. To substitute aspartic acid with alanine in *LKH1*, pNEO-*LKH1-KD* was amplified by PCR using KOD DNA polymerase (TOYOBO) with primers J2006 and J2007 and pNEO-*LKH1* as a template and subsequently digested with DpnI (Enzynomics) at 37°C for 1 h to digest pNEO-*LKH1*. DpnI-treated PCR products were transformed into competent cells. After sequencing with no errors, the SnaBI-digested pNEO-*LKH1-KD* was introduced into the *lkh1*Δ mutant (KW1451) by biolistic transformation. The pNEO-*LKH1-KD* integration was confirmed by diagnostic PCR, and the *LKH1-KD* allele expression level in the *lkh1*Δ mutant was measured by qRT-PCR analysis using *LKH1*-specific primers, J1692 and J1908.

### 2.5 Stress and antifungal drug susceptibility tests

*C. neoformans* strains were cultured in 2 mL liquid YPD medium at 30°C overnight. Strains were serially diluted 10-fold (1 to 10^4^) and spotted onto YPD medium containing diverse types of stress agents [bleomycin, methyl methanesulfonate (MMS), hydroxyurea (HU), 4-nitroquinoline 1-oxide (4-NQO), cisplatin, amphotericin B (AmpB), fluconazole (FCZ), caffeine, calcofluor white (CFW), congo-red (CR), sodium dodecyl sulfate (SDS), H_2_O_2_, diamide and menadione]. Strains that were spotted onto the YPD medium containing stress agents were incubated at 30°C for 2–5 days.

### 2.6 Total RNA isolation, cDNA synthesis, and quantitative RT-PCR

The strains were cultured in YPD medium for 16 h at 30°C and subcultured in fresh liquid YPD medium at 30°C until the optical density of the culture medium at 600 nm reached 0.6. The 50 ml of cells were harvested and pelleted by centrifugation for a basal time. The remaining cultures were treated with diverse agents. The cultures were further incubated and harvested at the indicated time points. The harvested samples were frozen in liquid nitrogen and lyophilized overnight. Total RNA was isolated using Easyblue (Intron, #17061), as previously described (Ko et al., 2009), and purified using an RNeasy Mini Kit (Qiagen, #74104) following the manufacturer’s instructions. cDNA was synthesized using the PrimeScript 1st strand cDNA synthesis kit (TAKARA, #6110A) using purified total RNAs as a template. qRT-PCR analysis was performed with the gene-specific primers listed in **Supplementary Table S2** using the CFX96 real-time PCR detection system (Bio-Rad). Relative target gene expression was determined using the 2^- Δ ΔCt^ method and *ACT1* expression was used for internal control (Livak and Schmittgen, 2001). Statistical analyses were performed using a one-way analysis of variance (ANOVA) with Bonferroni’s multiple comparison test (GraphPad, USA).

### 2.7 Intracellular reactive oxygen species measurement

*C. neoformans* strains were incubated overnight at 30°C. The cells were then centrifuged and washed with 1×PBS. The cell quantities were synchronized using a hemocytometer. Subsequently, synchronized cells were treated with menadione and harvested at each time point. Samples were fixed as previously described (Jung et al., 2012). Each sample was divided into two parts: one part was treated with 10 μM H2DCFDA, and the other was used as a control. All samples were incubated for 1 h in the dark at room temperature. After incubation, all samples were washed three times with 1 × PBS and divided into 96 Grenier plates and 96 flat black plates. Absorbance was measured at an optical density of 600 nm and fluorescence was measured at 488 and 522 nm.

### 2.8 Protein extraction and western blot analysis

WT (H99) and *lkh1*Δ strain were grown in a liquid YPD medium for 16 h at 30°C and subcultured in a fresh liquid YPD medium at 30°C until the optical density at 600 nm of the culture medium ranged between 0.7 and 0.8. The cell culture was harvested as a basal sample, and the remaining culture was treated with either caffeine (final concentration: 1 mg/mL). After treating the stress agent, 25 mL of the cell culture was harvested using an equal volume of ice-cold protein-stopping buffer (0.9% NaCl, 1 mM NaN_3_, 10 mM EDTA, and 50 mM NaF) at the indicated time points which are 0.5, 1, 2, and 3 h for caffeine treatment. The concentration of protein extracted from the cells was quantitatively measured using a bicinchoninic acid (BCA) kit (Thermo Fisher, #23227). A total of 50 µg of each protein was loaded onto a 10% Tris-glycine SDS-polyacrylamide gel and transferred to a nitrocellulose membrane. To detect phosphorylated Mpk1, a 1:4000 dilution of phosphor-p44/42 MAPK (Erk1/2) (Thr202/Tyr204) rabbit monoclonal antibody (Cell Signaling Technology, #4370S) was used. To ensure equal protein loading, we used 1/4000 dilution of a Hog1-specific rabbit polyclonal antibody (Santa Cruz, SC-9079). Protein levels were measured using a ChemiDoc (Bio-Rad).

For phosphatase assay, the Rad53-4×FLAG strain and Lkh1-4×FLAG strain were inoculated in YPD medium at 30°C overnight and subcultured into 200 mL YPD medium at an OD_600_ of 0.2 and incubated until OD_600_ reached approximately 0.8. We harvested 100 mL of the culture for the basal-time sample, and the remaining sample was treated with rapamycin or MMS and further incubated for 2 h at 30°C. The collected samples were divided into two halves for phosphate treatment. One half was resuspended in lysis buffer containing a phosphatase inhibitor cocktail, and the other half was resuspended in lysis buffer without a phosphatase inhibitor cocktail. The proteins extracted from each sample were quantified using a BCA kit and adjusted to the same protein concentration. Next, protein samples were treated with PMP buffer and 800 units of phosphatase (New England Biolabs, #P0753S) for 1 h at 30°C.

### 2.9 Mating analysis

The *C. neoformans* strains were cultured in a liquid YPD medium for 16 h at 30°C. Cell concentrations were calculated by hemocytometer, and the *MAT*α and *MAT* **a** cell concentrations were synchronized (10^7^ cells/mL). Equal amounts of *MAT*α and *MAT* **a** cells were mixed, spotted (5 μl) onto V8 mating medium, and grown in the dark at room temperature for 6 weeks. Filamentous growth was observed and photographed once a week using a microscope equipped with a SPOT Insight digital camera (Diagnostic Instrument, Inc.).

### 2.10 Ergosterol measurement

The ergosterol content of the strains was measured as described previously (Ko et al., 2009). The strains were inoculated in YPD medium at 30°C overnight and subcultured into 150 mL YPD medium at an OD_600_ of 0.2 and incubated until OD_600_ reached about 0.8. We harvested 50 mL of the culture for the basal-time sample, and the remaining sample was treated with FCZ (10 μg/mL) and further incubated until each time point at 30°C. The ergosterol content of the sample was calculated as follows: % ergosterol = [(OD_281.5_/290) × F]/pellet weight – [(OD_230_/518) × F]/pellet weight, where F is the ethanol dilution factor and 290 and 518 are the E values (in percentage per centimeter) determined for crystalline ergosterol and 24(28) dehydroergosterol, respectively (Arthington-Skaggs et al., 1999).

### 2.11 Intracellular ATP measurement

The amount of intracellular ATP was measured as described previously (Garcia et al., 2008; Price et al., 2011). WT, *lkh1*Δ, and *lkh1*Δ+*LKH1* strains were grown in liquid YPD medium for 16 h at 30°C and subcultured in fresh liquid YPD medium at 30°C until the optical density at 600 nm of the culture medium ranged between 0.7 and 0.8. Cultured samples were harvested and washed with PBS. Samples were resuspended in 30 mL PBS and 15 mL aliquots of each sample were centrifuged. Pellets for ATP analysis were resuspended in 1 mL of 50 mM HEPES (pH 7.7) and disrupted using a bead beater (Precellys) for 45 s. The disrupted samples were centrifuged at 5,000 rpm at 4°C for 10 min, and the supernatants were collected to determine ATP concentrations using an ATP bioluminescence assay kit (Sigma, #FLAA). Protein concentrations used to normalize the ATP concentration of each sample were measured in an aliquot of the supernatant from each sample using a BCA kit (Thermo Fisher, #23227).

### 2.12 Measurement of expression levels of genes involved in melanin and capsule productions

Quantitative measurement of *LAC* (*LAC1* and *LAC*2) genes expression was performed using a dopamine medium to induce melanin production. Strains were cultured in liquid YPD medium overnight at 30°C and subcultured in fresh liquid YPD medium and then further incubated until they reached an approximate OD_600_ of 0.6. Basal-level samples were harvested, and the remaining samples were washed three times using sterile water. After that, the remained cultures were resuspended in a dopamine medium and harvested after 4 h. To measure *CAP10*, *CAP59*, *CAP60*, and *CAP64*, strains were cultured in liquid YPD medium overnight at 30°C. The grown cells were subcultured in fresh liquid YPD medium at 30°C until the optical density of the culture medium ranged from 0.6 to 0.7. Next, 50 mL of the grown cells were harvested for the basal sample, and the remaining samples were washed three times using sterile water. After washing, the culture was resuspended in Littman’s medium and incubated for 4 h. Total RNA isolation and cDNA synthesis was performed as previously described (Ko et al., 2009). qRT-PCR analysis was performed using the gene-specific primers listed in **Supplementary Table S2**.

### 2.13 RNA-seq and data analysis

WT (H99) and *lkh1*Δ mutant were cultured in liquid YPD medium overnight at 30°C, subcultured in fresh liquid YPD medium, and then further incubated until they reached an approximate OD_600_ of 0.7–0.8. For basal samples, we collected 50 mL of grown cells and treated them with rapamycin (3 ng/mL) the remaining cells for 1 h. After 1 h, the cells were harvested and placed in liquid nitrogen. All samples were lyophilized and total RNA was isolated using Trizol reagent (Intron, #17061) and purified with an RNeasy mini kit (Qiagen, #74104) according to the manufacturer’s protocol. To ensure the reliability of the RNA-seq analysis, we prepared three biologically independent samples for each strain. Total RNA concentration was calculated using Quant-IT RiboGreen (Invitrogen, #R11490). To assess the integrity of the total RNA, samples were run on TapeStation RNA screen tape (Agilent, #5067-5576). Only high-quality RNA preparations (RIN greater than 7.0) were used for the RNA library construction. A library was independently prepared with 0.5 μg of total RNA for each sample by Illumina TruSeq Stranded Total RNA Library Prep Gold Kit (Illumina, Inc., San Diego, CA, USA, # 20020599). The first step in the workflow involved removing rRNA from the total RNA using the Ribo-Zero rRNA Removal Kit (Human/Mouse/Rat Gold) (Illumina, Inc., San Diego, CA, USA). Subsequently, the remaining mRNA was fragmented into small pieces using divalent cations at elevated temperatures. The cleaved RNA fragments were copied into first-strand cDNA using SuperScript II reverse transcriptase (#18064014; Invitrogen) and random primers. This was followed by the synthesis of second-strand cDNA using DNA Polymerase I, RNase H, and dUTP. These cDNA fragments then go through an end repair process, the addition of a single ‘A’ base, and then adapter ligation. The products were purified and enriched using PCR to create a final cDNA library. Libraries were quantified using KAPA Library Quantification kits for Illumina Sequencing platforms per the qPCR Quantification Protocol Guide (KAPA BIOSYSTEMS, #KK4854) and qualified using a TapeStation D1000 ScreenTape (Agilent Technologies, # 5067-5582). Indexed libraries were then subjected to Illumina NovaSeq (Illumina, Inc., San Diego, CA, USA), and paired-end (2×100 bp) sequencing was performed by Macrogen Inc. The RNA-seq data generated in this study were available from the Gene Expression Omnibus (GSE233612).

### 2.14 Virulence assay

SPF/VAF-confirmed inbred 6-week-old female BALB/cAnNCrlOri mice were purchased (ORIENT BIO Inc., Republic of Korea) and used in the experiment after acclimatization to the breeding environment for one week. For infection, the strains [WT (H99), *lkh1*Δ (KW1451), and *lkh1*Δ+*LKH1* (KW1633)] were inoculated in a fresh liquid YPD medium and cultured overnight in a 30°C shaking incubator. After adjusting the number to 5 × 10^5^ yeast cells, respiratory anesthesia was induced with isoflurane, and the strains were inhaled nasally. After infection, the condition of the mice was observed daily, and the survival rate of the mice was expressed as a percentage of survival. Statistical analysis was performed using Log-rank (Mantel-Cox) test using Graphpad Prism 9.5.1.

### 2.15 *In vitro* blood-brain barrier translocation assay

For the BBB translocation assay, human brain microvascular endothelial cells (hCMEC/D3 cell line; Merck & CO) were cultured as previously described (Vu et al., 2009; Santiago-Tirado et al., 2017; Jin et al., 2020; Lee et al., 2020). Briefly, 5×10^4^ hCMEC/D3 cells in EGM-2 medium (Lonza Group) were seeded on collagen (Corning, Inc)-coated 8 μm-porous membranes (BD Falcon). The day after seeding, the medium was replaced with fresh EGM™-2 medium supplemented with 2.5% human serum, and the cells were further grown for 4 days. A day before the inoculation of each strain, the medium was replaced with 0.5×diluted EGM™-2 medium (1.25% human serum), and the cells were maintained at 37°C and 5% CO_2_ incubator. The integrity of tight junctions between hCMEC/D3 cells was confirmed by measuring the transendothelial electrical resistance (TEER), which should be approximately 200 Ω per cm^2^. TEER was measured using the Epithelial Volt per Ohm Meter (EVOM2 device, World Precision Instruments). We added 5×10^5^ cells of each strain to 500 μl of PBS, inoculated them onto the top of the porous membranes, and incubated them for 24 h at 37°C in a 5% CO_2_ incubator. The number of yeast cells passing through the porous membrane was determined by counting the CFU. Tight junctions between the hCMEC/D3 cells were measured using the EVOM2 device before and after inoculation with the strains.

### 2.16 Histochemistry assay

For tissue staining, lungs were obtained from mice on day 16 after infection, weighted, and fixed in a 3.7% formaldehyde solution. After complete fixation, the tissues were dehydrated and clarified using xylene before being embedded in paraffin blocks. Tissue sections with a thickness of 5 μm were cut and stained with Grocott’s Methenamine Silver (GMS) Stain Kit (Abcam, ab287884) or Periodic Acid Schiff (PAS) Stain Kit (Mucin Stain) (Abcam, ab150680), per the manufacturer’s protocol. Slide images were captured with Primostar 3 (ZEISS, Germany) at final magnifications of 100 × and 200 ×.

### 2.17 Flocculation and adhesion assays

The flocculation assay was performed as previously described (Cramer et al., 2006). Briefly, *C. neoformans* strains were cultured in liquid YPD medium for 16 h at 30°C. Subsequently, the grown cells were normalized to an OD_600_ of 1.5. The OD_600_ of each cell suspension was then measured for a duration of 4 h. Three independent biological replicates were conducted, and error bars indicate the standard error of the means (S.E.M). The adhesion assay was performed following the protocol outlined in the previous study (Lee et al., 2014). Strains were cultured in liquid YPD medium for 16 h at 30°C. Next, the 5 μl of the grown cells were spotted onto filament agar plates and incubated for 5 days at 22°C. Adherence was assessed by washing the plate under flowing distilled water for 20 sec.

### 2.18 Flow cytometry analysis

For flow cytometry analysis, we prepared WT (H99) and *lkh1*Δ mutant strains, as previously described (Choi et al., 2024). Briefly, WT and *lkh1*Δ mutant strains were cultured in a liquid YPD medium for 16 h. Next, each strain was grown to an optical density at 600 nm of 0.8 and washed with phosphate-buffered saline (PBS). After cells were fixed with ethanol, cells were washed with PBS containing 1% and 0.5% bovine serum albumin (BSA). Subsequently, the cells were treated with RNase for 30 min at 37°C and stained with propidium iodide staining buffer for 2 h at room temperature in the dark. After washing, fluorescence was assessed using a BD FACS symphony A5, capturing 10,000 events per sample.

### 2.19 Measurement of chitin contents

For chitin staining, the strains were cultured in YPD medium for 16 h at 30°C and subsequently subcultured in fresh liquid YPD medium until the optical density of the culture medium at 600 nm reached 0.8. A 50 mL aliquot of cells was harvested and pelleted by centrifugation to establish a basal time point. The remaining cultures were treated with 1.0 mg/mL of caffeine and further incubated before being harvested after 12 hours. Harvested samples were fixed following previously established methods (Jung et al., 2012). Fixed samples were mixed with an equal volume of calcofluor white solution (25 μg/mL) and incubated for 30 min at room temperature in darkness. Subsequently, the cells were washed twice with PBS and mixed with mounting buffer (Biomeda, cat. NO #M01) on slide glass for immobilization, followed by an additional 30 min incubation. Each sample was then photographed using fluorescence microscopy (BX51; Olympus, Tokyo, Japan), and fluorescence intensity was measured in a minimum of 50 individual cells using the ImageJ/Fiji software following previous methods (Kim et al., 2021).

## 3 Results

### 3.1 Identification of LAMMER kinase, Lkh1, in *C. neoformans*

**Figure 1:**
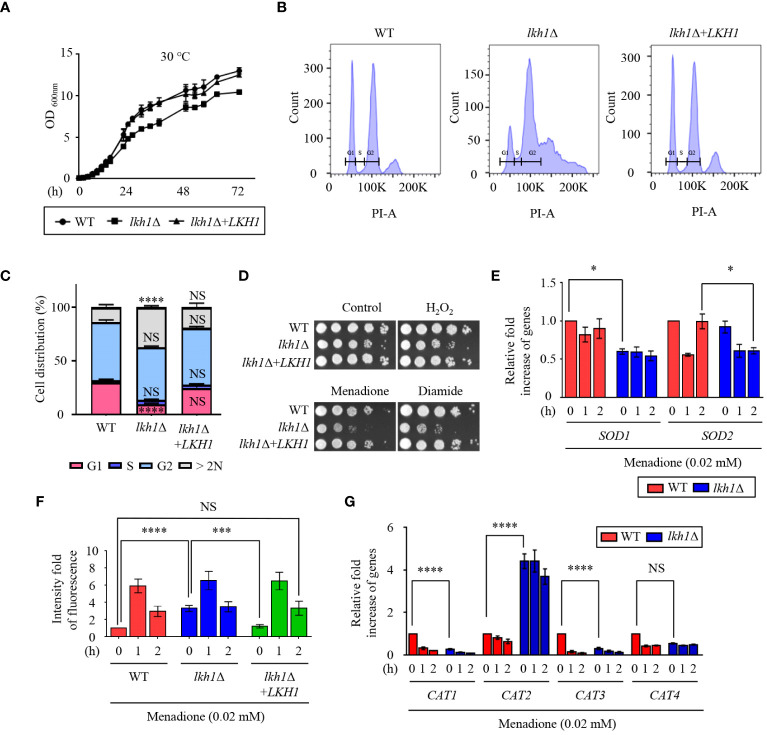
Lkh1 regulated growth and oxidative stress response. **(A)** WT (H99), *lkh1*Δ (KW1451), and *lkh1*Δ+*LKH1* (KW1633) strains were grown in liquid YPD medium for 16 h at 30°C and subcultured in fresh liquid YPD medium at 30°C. Cell density was measured at an optical density of 600 nm every 2 (h) **(B, C)** Histogram of cell count in WT and *lkh1*Δ mutant by propidium iodide using flow cytometry. The provided images represent data from three biological replicates **(B)**. Population proportion of cells were quantified. One-way ANOVA was used to dertermine statistical differences among strains (NS: not significant; * *P* < 0.05; *** *P* < 0.001; **** *P* < 0.0001) **(D)** WT (H99), *lkh1*Δ (KW1451), and *lkh1*Δ+*LKH1* (KW1633) strains were cultured in liquid YPD medium overnight at 30°C, 10-fold serially diluted and spotted on YPD medium containing 0.02 mM menadione, 2 mM diamide, or 2 mM H_2_O_2_. **(E, G)** Expression levels of *SOD* genes and *CAT* genes were verified by qRT-PCR analysis using cDNA of WT (H99) and *lkh1*Δ strains with or without treatment of menadione (0.02 mM). Three independent biological experiments with duplicate technical replicates were performed. Error bars indicate the standard error of the mean (S.E.M). The statistical significance of the differences was determined by one-way ANOVA with Bonferroni’s multiple-comparison test. (NS: not significant; * *P* < 0.05; *** *P* < 0.001; **** *P* < 0.0001) **(F)** Intracellular ROS levels of WT, *lkh1*Δ, and *lkh1*Δ+*LKH1* strains treated with or without MD (0.02 mM) were measured using H2DCFDA.

To identify the LAMMER kinase ortholog in *C. neoformans*, BLAST searches were performed using the *S. cerevisiae* Kns1 protein sequence as a query. We identified a single gene (CNAG_00683) with high scores and e-values (score: 294; e-value: 3e-89). The putative LAMMER kinase harbors the conserved motif ‘EHLAMMERILG’ sequence similar to other fungal LAMMER kinases. Therefore, we named CNAG_00683 as Lkh1 in *C. neoformans*. Growth defect is a conserved phenotype observed in *lkh1*Δ mutants of other fungi (Kang et al., 2010; Park et al., 2011; Kang et al., 2013). To ascertain whether the *Cryptococcus lkh1*Δ mutant exhibited growth defect, we generated the *lkh1*Δ mutant and its complemented strain and monitored their growth at 30°C and 37°C. We found that the *Cnlkh1*Δ mutant displayed a growth defect compared to WT and complemented strain at both temperatures (**Figure 1A** and **Supplementary Figure S2A**). ATP synthesis is essential for fungal cell growth (Li et al., 2021; Vestergaard et al., 2022). To establish a relationship between the growth defects of the *lkh1*Δ mutant and intracellular ATP levels, we measured the intracellular ATP content in each strain. The ATP levels in the *lkh1*Δ mutant were indistinguishable from those in the WT, indicating that ATP content was not associated with the growth defects observed in the *lkh1*Δ mutant (**Supplementary Figure S2B**). Given that Lkh1 is involved in cell cycle progression by phosphorylating Rum in fission yeast (Yu et al., 2013) and *lkh1*Δ mutant showed slow growth in *C. neoformans*, we conducted a fluorescence-activated cell sorting (FACS) analysis to investigate whether CnLkh1 plays a role in cell cycle progression. We found that the WT strain showed regular G1, S, and G2 phases, whereas abnormal cell cycle patterns were observed in the *lkh1*Δ mutant. The *lkh1*Δ mutant exhibited a decreased G1 phase and increased G2 phase compared to the WT strain. Furthermore, the *lkh1*Δ mutant exhibited increased DNA content beyond 2N (**Figures 1B, C**, and **Supplementary Figure S2C**). Morphological analysis of the *lkh1*Δ mutant revealed that some of the cells were not physically separated compared to WT and its complemented strain (**Supplementary Figure S2D**). Therefore, these data suggest that *C. neoformans* Lkh1 is also involved in the cell cycle progression akin to other fungi.

### 3.2 Lkh1 was involved in oxidative stress response by regulating intracellular ROS levels

A previous study demonstrated that strains with deleted *LKH1* showed growth defects in response to hydrogen peroxide (Park et al., 2003). To demonstrate the involvement of CnLkh1 in the oxidative stress response, we conducted a phenotypic analysis. The *lkh1*Δ mutant showed increased susceptibility to menadione and diamide, but not to hydrogen peroxide (**Figure 1D**). Menadione serves as a general superoxide generator, and Sods protect cells from the toxic effects of reactive oxygen species, which are mediated by the dismutation of superoxide radicals into hydrogen peroxide and oxygen (Miller and Britigan, 1997). To examine whether Lkh1 regulates the expression of *SOD* genes, we evaluated the expression of *SOD* genes in the WT and *lkh1*Δ mutant under menadione treatment. The *lkh1*Δ mutant exhibited diminished *SOD1* expression compared to the WT, while the *SOD2* expression level in the *lkh1*Δ mutant was lower than that in WT at 2 h post-menadione treatment (**Figure 1E**). Next, we sought to determine if the reduced *SOD1* expression levels in the *lkh1*Δ mutant corresponded to changes in intracellular ROS levels. To address this, we quantified intracellular ROS levels in WT, *lkh1*Δ, and *lkh1*Δ+*LKH1* strains using H2DCFDA. We observed that the intracellular ROS level in the *lkh1*Δ mutant was higher than that in WT at the basal level. However, ROS productions in the WT and *lkh1*Δ mutant were indistinguishable under menadione treatment (**Figure 1F**). In *S. pombe*, expression levels of the catalase gene, *CTT1*, are markedly reduced in the *lkh1*Δ mutant compared to those in WT (Park et al., 2003). To investigate whether Lkh1 is involved in regulating catalase genes, we assessed the expression levels of four catalase genes (*CAT1*, *CAT2*, *CAT3*, and *CAT4*) in the presence of menadione. The expression levels of *CAT1* and *CAT3* genes in the *lkh1*Δ mutant were reduced compared to those in the WT under basal conditions, whereas the *CAT2* gene expression levels in the *lkh1*Δ mutant were significantly higher than those in the WT, regardless of the presence or absence of menadione (**Figure 1G**). Collectively, these results suggested that Lkh1 governs the oxidative stress response by modulating the expression of *SOD* and *CAT* genes.

### 3.3 Lkh1 controlled DNA damage response in both Rad53-dependent and -independent manners

**Figure 2:**
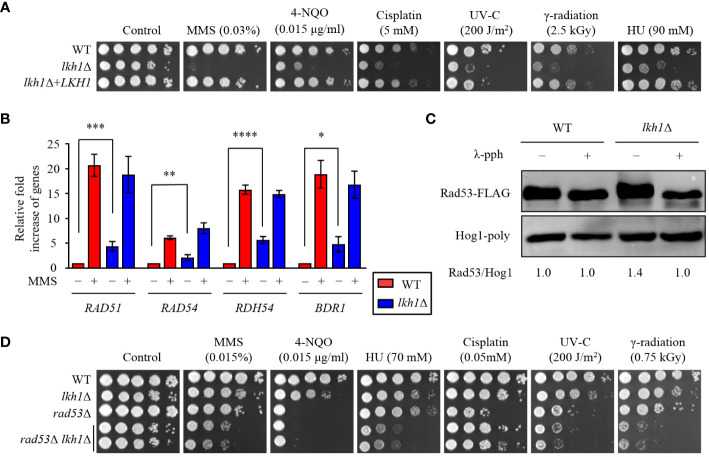
Lkh1 regulated DNA damage response. **(A)** WT (H99), *lkh1*Δ (KW1451), and *lkh1*Δ+*LKH1* (KW1633) strains were cultured in a liquid YPD medium overnight at 30°C, 10-fold serially diluted and spotted on YPD medium containing the indicated concentration of DNA damage insults. For UV-C and γ-radiation tests, the serially diluted cells were spotted onto YPD medium and exposed to the indicated dose of UV-C (200 J/m^2^) or γ-radiation (2.5 kGy). Next, cells were further incubated at 30 °C and photographed daily for 2-4 days. **(B)** Expression levels of the *BDR1* gene and Rad53-Bdr1 pathway downstream genes were verified by qRT-PCR analysis using cDNA of WT and *lkh1*Δ mutant with or without treating MMS (0.02%). Three independent biological experiments with duplicate technical replicates were performed. Error bars indicate S.E.M. Statistical significances of the differences were determined by one-way ANOVA with Bonferroni’s multiple-comparison test. (* *P* < 0.05; ** *P* < 0.01; *** *P* < 0.001; **** *P* < 0.0001) **(C)** Phosphorylation of Rad53 was monitored by analysis of the reduced electrophoretic migration using western blotting with anti-FLAG antibody. The Rad53-4×FLAG strain (YSB3806) and *lkh1*Δ Rad53-4×FLAG (KW1806) were grown to the mid-logarithmic phase and the cell extract was incubated at 30°C for 1 h with or without λ-phosphatase (PPase). **(D)** WT (H99), *lkh1*Δ (KW1451), *rad53*Δ (YSB3785), and *rad53*Δ *lkh1*Δ (KW1610 and KW1612) strains were cultured in a liquid YPD medium overnight at 30°C, 10-fold serially diluted and spotted on YPD medium containing the indicated concentration of DNA damage insults. For UV-C and γ-radiation tests, the serially diluted cells were spotted onto a YPD medium and exposed to the indicated dose of UV-C or γ-radiation. Next, cells were further incubated at 30°C and photographed daily for 2-4 days.

Previous studies have reported a critical role for *U. maydis* Lkh1 in maintaining genome stability and facilitating DNA repair processes (de Sena-Tomás et al., 2015). To demonstrate the requirement of Lkh1 in DNA damage response, survival tests were conducted under DNA damage stress conditions. Consistent with observation in the *U. maydis lkh1*Δ mutant, the *lkh1*Δ mutant exhibited a significant growth defect in response to various types of DNA damage stress (**Figure 2A**). In *C. neoformans*, the Rad53-Bdr1 pathway plays a central role in the response to DNA damage stress by regulating the expression of DNA repair genes (Jung et al., 2016, 2019, 2021). To demonstrate that Lkh1 controls the DNA damage response via the Rad53-Bdr1 pathway, we measured the expression levels of *BDR1* and its downstream genes. Notably, the *lkh1*Δ mutant displayed inherently higher expression levels of *BDR1*, *RAD51*, *RAD54*, and *RDH54* compared to the WT, whereas induction levels upon MMS treatment were similar between WT and *lkh1*Δ strains (**Figure 2B**). This result led us to compare Rad53 phosphorylation levels in WT and *lkh1*Δ strains because Rad53 phosphorylation induces the expression of DNA repair genes. To compare Rad53 phosphorylation, we constructed *lkh1*Δ mutant strains in the Rad53-4×FLAG background and monitored Rad53 phosphorylation in both WT and *lkh1*Δ mutant. We observed that Rad53 was more phosphorylated in *lkh1*Δ mutant compared to WT under a basal condition (**Figure 2C**). These findings indicate that DNA damage responses in the *lkh1*Δ mutant are more impaired than those in WT at the basal condition.

Because Lkh1 controlled DNA damage response in both Rad53-dependent and independent manner, we hypothesized that *rad53*Δ *lkh1*Δ double mutants show more pronounced growth defect under DNA damage stress compared to the individual *lkh1*Δ and *rad53*Δ mutant. To validate this hypothesis, we constructed two additional independent *rad53*Δ *lkh1*Δ double mutants and performed phenotypic analyses using them. Expectedly, the *rad53*Δ *lkh1*Δ double mutants were more susceptibility to DNA damage insults compared to the individual *lkh1*Δ and *rad53*Δ mutant (**Figure 2D**). Therefore, Lkh1 plays an important role in the DNA damage response in both Rad53-dependent and -independent manners.

### 3.4 Lkh1 controlled cell wall integrity in an Mpk1-dependent and -independent manner

**Figure 3:**
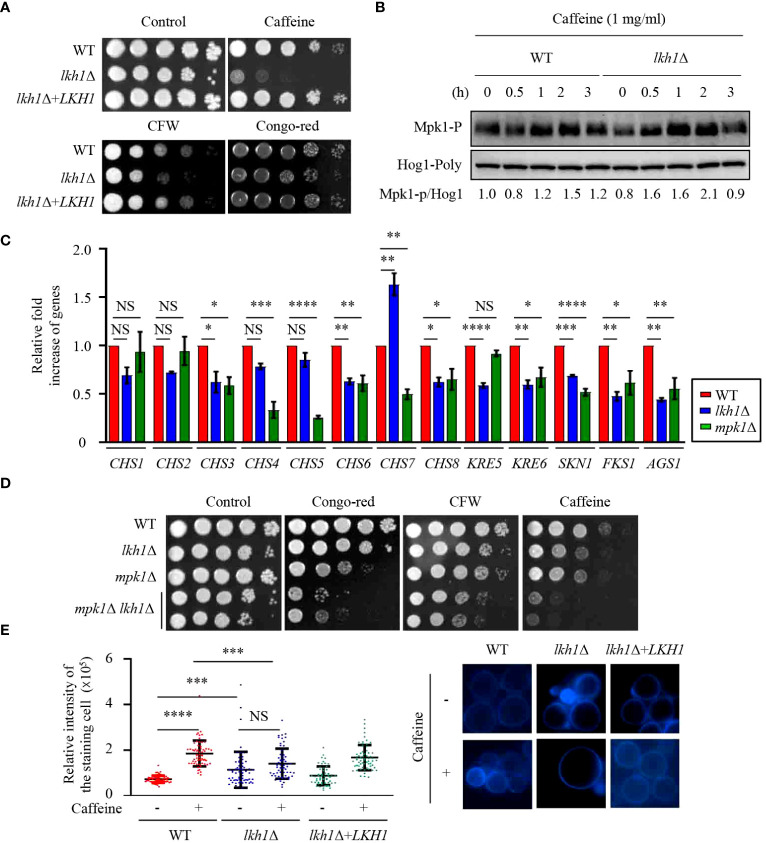
Lkh1 was involved in the cell wall damage stress. **(A)** WT (H99), *lkh1*Δ (KW1451), and *lkh1*Δ+*LKH1* (KW1633) strains were cultured in a liquid YPD medium overnight at 30°C, 10-fold serially diluted and spotted on a YPD medium containing 60 mg/mL CFW, 1 mg/mL caffeine, or 1% congo-red. **(B)** Mpk1 phosphorylation was monitored using western blotting with an anti-phospho-Erk1 antibody. The WT (H99) and *lkh1*Δ strains were grown to the mid-logarithmic phase and then treated with caffeine (1 mg/mL) for 3 h. Next, total protein was extracted from each strain for immunoblot analysis. **(C)** Expression levels of chitin synthesis-related genes and glucan synthesis-related genes were verified by qRT-PCR analysis using cDNA of WT (H99) and *lkh1*Δ strains. Three independent biological experiments with duplicate technical replicates were performed. Error bars indicate S.E.M. Statistical significances of the differences were determined by one-way ANOVA with Bonferroni’s multiple-comparison test. (* *P* < 0.05; ** *P* < 0.01; **** *P* < 0.0001; NS: not significant) **(D)** WT (H99), *lkh1*Δ (KW1451), *mpk1*Δ (YSB3814), and *mpk1*Δ *lkh1*Δ (KW1858 and KW1860) strains were cultured in liquid YPD medium overnight at 30°C, 10-fold serially diluted and spotted on a YPD medium containing 1 mg/mL CFW, 0.3 mg/mL caffeine, or 0.04% congo-red. **(E)** Chitin staining with CFW. The quantitative fluorescence intensity of 50 individual cells of each strain were measured suing ImageJ/Fiji software. The error bar indicates the standard deviations (SD). The statistical significance of the differences was determined by one-way ANOVA using Bonferroni’s multiple-comparison test. (*** *P* < 0.001; **** *P* < 0.0001; NS: not significant).

In *C. albicans*, deletion of *KNS1* results in growth defects in response to cell stress agents and alterations in cell wall composition (Park and Park, 2011; Choi et al., 2014). Similar to the *kns1*Δ mutant in *C. albicans*, the *lkh1*Δ mutant showed growth defects in response to cell wall stress agents, such as caffeine, calcofluor white, and congo-red (**Figure 3A**). In *C. neoformans*, the Mpk1 MAPK pathway plays a central role in regulating cell wall integrity through phosphorylation in response to cell wall stress agents (Kraus et al., 2003). To ascertain the potential involvement of Lkh1 in Mpk1 phosphorylation during cell wall stress, we monitored the phosphorylation status of Mpk1 in *lkh1*Δ mutant under cell wall stress conditions. We observed that Mpk1 phosphorylation levels in *lkh1*Δ mutant were slightly increased than those in WT in the presence of caffeine (**Figure 3B**).

The cell wall of *C. neoformans* is composed of α- and β-glucan as well as chitin. *C. neoformans* harbors eight putative chitin synthase encoding genes, seven β-glucan synthesis-related genes, and one α-glucan synthase encoding gene (Thompson et al., 1999; Reese and Doering, 2003; Banks et al., 2005; Reese et al., 2007; Wang et al., 2018). Given that the *lkh1*Δ mutant exhibited growth defects in response to cell wall stress agents, we wondered whether Lkh1 controls the expression of genes involved in cell wall biosynthesis. To test this hypothesis, we measured the expression of chitin synthase-associated genes. The expressions of *CHS3*, *CHS6*, and *CHS8* in the *lkh1*Δ mutant were reduced compared to WT, while the expression of *CHS7* in the *lkh1*Δ mutant was elevated relative to the WT (**Figure 3C**). β-1,6-glucan is the predominant polysaccharide in the *C. neoformans* cell wall compared to β-1,3-glucan (Zaragoza et al., 2009; Gilbert et al., 2010). To confirm Lkh1 involvement in β-glucan synthesis in *C. neoformans*, we measured the expression levels of related genes. The expressions of *KRE6* (β-1,6-glucan synthase-related gene), *FKS1* (encoding β-1,3-glucan synthase), and *ASG1* (α-1,3-glucan synthase) were reduced in both *mpk1*Δ mutant and *lkh1*Δ mutant compared to WT. The expression of the *KRE5* (β-1,6-glucan synthase-related gene) in the *lkh1*Δ mutant was reduced compared to WT but remained unaffected in the *mpk1*Δ mutant (**Figure 3C**). Next, to reinforce the notion that Lkh1 is involved in cell wall integrity in an Mpk1-independent manner, we constructed *mpk1*Δ *lkh1*Δ double mutants and subjected them to a cell wall stress assay. Expectedly, *mpk1*Δ *lkh1*Δ double mutants showed significantly higher susceptibility to cell wall damage agents compared to the individual *lkh1*Δ and *mpk1*Δ mutants (**Figure 3D**). Given that expression levels of genes related to chitin synthesis were diminished in *lkh1*Δ mutant compared to those in WT, we quantitatively measured chitin levels in WT and *lkh1*Δ mutant in the presence or absence of caffeine. Under basal conditions, the chitin content in the *lkh1*Δ mutant was higher than those in WT and its complemented strain. However, in the presence of caffeine, the chitin content in WT and *lkh1*Δ+*LKH1* strain increased, whereas that in the *lkh1*Δ mutant was not enhanced to the same extent as in WT (**Figure 3E**). Collectively, these findings suggest that Lkh1 controls the cell wall stress response in an Mpk1-dependent and -independent manner.

### 3.5 Lkh1 was involved in azole drug resistance regardless of ergosterol levels

**Figure 4:**
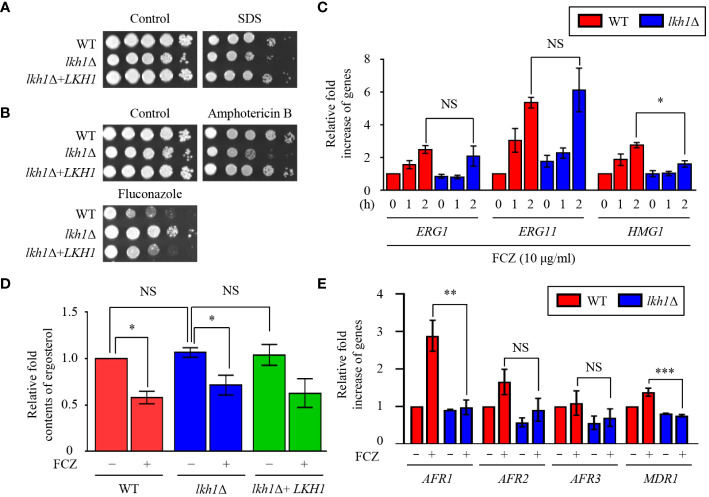
Lkh1 was involved in antifungal drug resistance. **(A, B)** WT (H99), *lkh1*Δ (KW1451), and *lkh1*Δ+*LKH1* (KW1633) strains were cultured in a liquid YPD medium overnight at 30°C, 10-fold serially diluted and spotted on YPD medium containing 0.02% SDS, 1.5 μg/mL amphotericin B, 14 μg/mL fluconazole. **(C, E)** Expression levels of ergosterol synthesis-related genes and ABC transporter genes were verified by qRT-PCR analysis using cDNA of WT (H99) and *lkh1*Δ strains with or without treatment of fluconazole (16 μg/mL). Three independent biological experiments with duplicate technical replicates were performed. Error bars indicate S.E.M. Statistical significances of the differences were determined by one-way ANOVA with Bonferroni’s multiple-comparison test. (* *P* < 0.05; ** *P* < 0.01; *** *P* < 0.001; NS: not significant) **(D)** The relative ergosterol contents of WT, *lkh1*Δ, and *lkh1*Δ+*LKH1* strains were assessed with or without fluconazole treatment.

The fungal cell membrane is a key target for antifungal drugs, primarily because of its specific fungal components such as ergosterol. Azole drugs inhibit ergosterol biosynthesis by binding to Erg11 and inducing the accumulation of 14-methylated sterols, compromising cell membrane integrity (Joseph-Horne et al., 1995). Amphotericin B interacts with the lipid bilayer membrane, induces pore formation, and increases cell permeability (Joseph-Horne et al., 1995). Given the observed susceptibility of the *lkh1*Δ mutant to cell membrane destabilizer (**Figure 4A**), an antifungal drug test was conducted. The *lkh1*Δ mutant showed susceptibility to amphotericin B whereas it exhibited resistance to fluconazole (**Figure 4B**). This contrasting response to azole and polyene drugs suggests a potential role of Lkh1 in the regulation of ergosterol biosynthesis. To test this hypothesis, we measured the expression levels of *ERG1*, *ERG11*, and *HMG1*, which participate in the rate-limiting step of ergosterol biosynthesis (Veen et al., 2003). We observed that expression levels of *ERG1* and *ERG11* in *lkh1*Δ mutant were similar to those in WT. However, the induction of *HMG1* in the *lkh1*Δ mutant was slightly lower than compared to the WT (**Figure 4C**). Although Lkh1 did not appear to be critical for the regulation of ergosterol biosynthesis genes, the ergosterol content was determined to further elucidate the regulatory role of Lkh1. Similar to expression levels of ergosterol biosynthesis genes, the ergosterol contents in the *lkh1*Δ were indistinguishable from those in WT (**Figure 4D**). In addition to ergosterol biosynthesis, several studies have demonstrated that ABC transporters are also critical for the acquisition of azole resistance in *C. neoformans* (Sanguinetti et al., 2006; Chang et al., 2018). To investigate whether Lkh1 regulates ABC transporters, we measured expression levels of these genes under fluconazole treatment. Contrary to our expectation, expression levels of ABC transporter genes such as *AFR1* and *MDR1* in *lkh1*Δ mutant were lower than those in WT under fluconazole treatment. However, the expression levels of *AFR2* and *AFR3* were similar in both *lkh1*Δ and WT strain in the presence of fluconazole (**Figure 4E**). Collectively, these findings suggest that Lkh1 controls azole resistance in an ergosterol biosynthesis-and ABC transporters-independent manners.

### 3.6 Deletion of *LKH1* decreased sexual differentiation and agar adherence and increased flocculation

**Figure 5:**
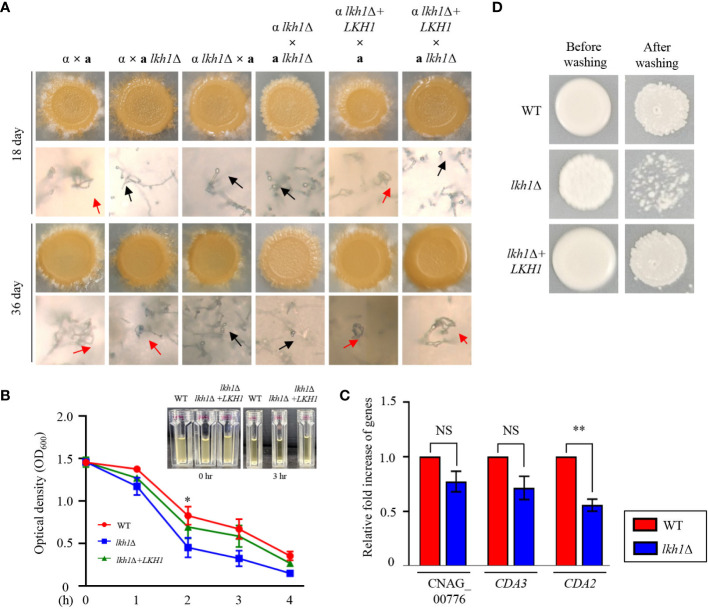
Lkh1 was required for basidiospores formation in *C neoformans.* **(A)** Serotype A *MAT*α and *MAT***a** strains were co-cultured on a V8 medium (pH 5.0) for 5 weeks at room temperature in the dark: WT α × WT **a** (H99 and KN99), WT α × **a** *lkh1*Δ (H99 and KW1529), α *lkh1*Δ × **a** (KW1451 and KN99), α *lkh1*Δ × **a** *lkh1*Δ (KW1451 and KW1529), α *lkh1*Δ+*LKH1* × **a** (KW1633 and KN99), and α *lkh1*Δ+*LKH1* × **a** *lkh1*Δ (KW1633 and KW1529). The images were photographed after 18 days and 36 days. The black arrows and red arrows indicate basidium and basidiospores, respectively. **(B)** WT, *lkh1*Δ, and *lkh1*Δ+*LKH1* strains were cultured in a liquid YPD medium, and each strain was normalized to an OD_600_ of 1.5. The OD_600_ was measured to determine flocculation at the indicated time points. The error bars indicate the standard error of the means (S.E.M). **(C)** The expression levels of adhesion-related genes in *lkh1*Δ mutant. Three independent biological experiments with duplicate technical replicates were performed. Error bars indicate the S.E.M. The statistical significance of the differences was determined by one-way ANOVA with Bonferroni’s multiple-comparison test. (NS: not significant; * *P* < 0.05; ** *P* < 0.01) **(D)** The same concentration of each strain was spotted on filament agar. After 5 days of incubation at room temperature, cells were washed with flowing water, followed by a subsequent incubation at 30°C for 2 days.

Deletion of *LKH1* results in reduced meiotic recombination in *U. maydis* and LkhA plays a pivotal role in the sexual development of *Aspergillus nidulans* (de Sena-Tomás et al., 2015, Kang et al., 2013). To demonstrate whether Lkh1 in *C. neoformans* is indispensable for sexual differentiation, we generated an *LKH1* deletion strain in a *MAT* **a** background and performed a mating assay. In unilateral crosses with the wild-type strains (H99 or KN99**a** strains) and *lkh1*Δ mutants, there was a noticeable delay in basidiospores production. However, basidiospores failed to form in bilateral crosses between the *MAT*α *lkh1*Δ mutant and *MAT* **a** *lkh1*Δ mutant (**Figure 5A**). These results indicate that Lkh1 is required for basidiospore formation in *C. neoformans*. Previous studies have reported that Lkh1 is involved in adhesion and flocculation in other fungi (Kim et al., 2001; Lim et al., 2021). Therefore, we performed flocculation and adhesion assays using WT, *lkh1*Δ, and *lkh1*Δ+*LKH1* complemented strains. We found that the *lkh1*Δ mutant exhibited quicker settling out of suspension than WT and *lkh1*Δ+*LKH1* strains (**Figure 5B**). In *C. neoformans*, mannoproteins (MPs) play a role in adhesion to host cells (Teixeira et al., 2014; Lee et al., 2023). Supporting this result, the expression level of adhesion-related gene *CDA2* in *lkh1*Δ mutant was reduced compared to that in WT (**Figure 5C**). This result indicates that Lkh1 plays a negative role in flocculation. Contrary to flocculation, the *lkh1*Δ mutant exhibited reduced agar adherence compared to the WT strain (**Figure 5D**).

### 3.7 Tor1 was an upstream regulator of Lkh1 in *C. neoformans*

**Figure 6:**
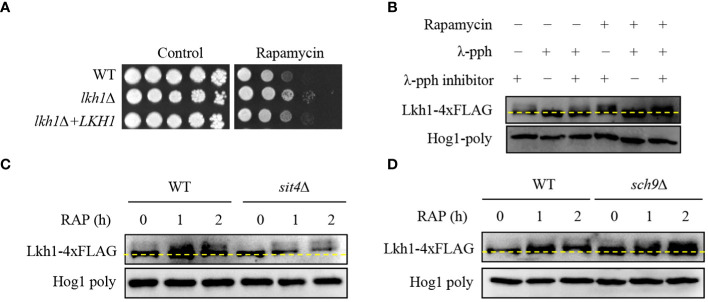
Phosphorylation of Lkh1 in a TOR1-dependent manner. **(A)** WT (H99), *lkh1*Δ (KW1451), and *lkh1*Δ+*LKH1* (KW1633) strains were cultured in a liquid YPD medium overnight at 30°C, 10-fold serially diluted and spotted on a YPD medium containing rapamycin (9 μg/mL). **(B–D)** Lkh1 phosphorylation was monitored using western blotting with an anti-FLAG antibody. The Lkh1-4×FLAG strain (KW1755), *sit4*Δ Lkh1-4×FLAG strain (KW2058), and *sch9*Δ Lkh1-4×FLAG strain (KW2091) were grown to the mid-logarithmic phase and then treated with rapamycin (3 ng/mL) for 2 h. The cell extract was incubated at 30°C for 1 h with or without λ-phosphatase (PPase) and PPase inhibitor.

In *S. cerevisiae*, the TOR signaling pathway regulates ribosome and tRNA synthesis through the negative regulation of LAMMER kinase (Lee et al., 2012). To reveal the relationship between Lkh1 and Tor1, we conducted a spotting assay using rapamycin, a Tor1 target. We observed *lkh1*Δ mutant showed resistance to rapamycin compared to WT (**Figure 6A**), suggesting that Lkh1 negatively regulates rapamycin resistance. To elucidate whether *C. neoformans* Lhk1 is phosphorylated by Tor1 kinase, we constructed strains containing *LKH1-4×FLAG* and confirmed the functionality of the Lkh1-4×FLAG fusion protein (**Supplementary Figure S3**). Next, we monitored the shift of Lkh1-4×FLAG in the presence of rapamycin. We found that Lkh1 was a slight upshift following rapamycin treatment (**Figure 6B**). To further demonstrate that the upshift in Lkh1 was caused by phosphorylation rather than by increased Lkh1 production, we compared the protein mobility of Lkh1 in the presence or absence of λ-phosphatase or phosphatase inhibitors. Notably, the mobility shift of Lkh1 was abolished in the presence of λ-phosphatase under untreated conditions, indicating that *C. neoformans* Lkh1 is intrinsically phosphorylated under basal conditions. In both the rapamycin and the λ-phosphatase treatments, the abolishment of the Lkh1 mobility shift was even more apparent (**Figure 6B**).

The fact that Lkh1 was phosphorylated under TOR kinase inhibition indicates that TOR-dependent kinases and phosphatases directly affect Lkh1 phosphorylation. In *S. cerevisiae*, the TOR kinase negatively regulates Sit4 phosphatase by facilitating complex formation between Tap42 and Sit4 (Di Como and Arndt, 1996). To demonstrate whether Sit4 is required for Lkh1 phosphorylation, we constructed a *sit4*Δ mutant in the background of the Lkh1-4×FLAG strain and monitored the Lkh1 protein shift. The data revealed that the Lkh1 protein in the *sit4*Δ mutant exhibited an increased mobility pattern compared to that in WT under both the absence and presence of rapamycin (**Figure 6C**). These data indicate that Sit4 indirectly influences Lkh1 phosphorylation. In *S. cerevisiae*, the TOR kinase directly phosphorylates the ACK kinase Sch9 (Urban et al., 2007). AKT, an ortholog of Sch9 in yeast, phosphorylates CLK1, CLK2, and murine LAMMER kinases (Song et al., 2023). To reveal whether Sch9 is required for Lkh1 phosphorylation, we constructed an *sch9*Δ mutant in the background of the Lkh1-4×FLAG strain. We observed that the Lkh1 protein in the *sch9*Δ mutant showed a significant upshift pattern compared to that of WT under both the absence and presence of rapamycin conditions (**Figure 6D**). This suggests that Sch9 is indirectly implicated in Lkh1 phosphorylation in an opposite manner to that of Sit4. Therefore, the Tor1-dependent pathway is responsible for Lkh1 phosphorylation.

### 3.8 Function of Lkh1 was entirely dependent on its kinase activity

**Figure 7:**
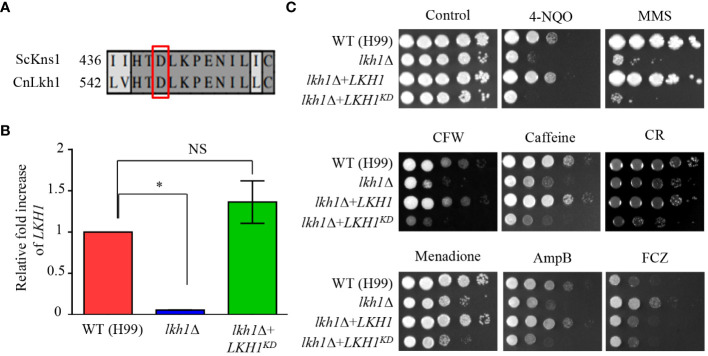
Kinase activity of Lkh1 was required for Lkh1 function. **(A)** Sequence alignment of the catalytic region for the kinase activity of *S. cerevisiae* Kns1 and *C neoformans* Lkh1. **(B)** The *LKH1* expression level in the *LKH1^KD^* strain. The qRT-PCR analysis was performed with cDNA synthesized from WT (H99), *lkh1*Δ (KW1451), and *LKH1^KD^* (KW1958) strains grown to the mid-log phase. Error bars indicate standard deviation. The statistical significance of difference was determined by one-way analysis of variance with Bonferroni’s multiple comparison tests. (* *P* < 0.05 and NS: non-significant) **(C)** WT (H99), *lkh1*Δ (KW1451), *lkh1*Δ+*LKH1* (KW1633), and *LKH1^KD^* (KW1958) strains were cultured in liquid YPD medium overnight at 30°C, 10-fold serially diluted and spotted on YPD medium containing stress-inducing agents. For the UV-C resistance test, the serially-diluted cells were spotting onto the YPD medium, and the indicated dose of UV-C was exposed. The plates were further incubated at 30°C and daily photographed for 2-4 days.

In *S. cerevisiae*, the Asp (D440) residue is required for the kinase activity of Kns1, and kinase-dead alleles decrease the Kns1 auto-phosphorylation (Lee et al., 2012). To assess the significance of the kinase activity of CnLkh1 for its functionality, we constructed the Lkh1 kinase-dead allele (*LKH1^D546A^*) and introduced it into the *lkh1*Δ mutant (**Figure 7A**). We confirmed the *LKH1^D546A^* allele expression level in the *lkh1*Δ mutant was similar to that in the WT (**Figure 7B**). Next, we observed that the resistance of *LKH1^D546A^* strains in response to various stress inducers except for fluconazole was similar to that of the *lkh1*Δ mutant (**Figure 7C**). These findings suggest that most Lkh1 functions are controlled by the kinase activity of Lkh1, except for regulating resistance to fluconazole.

### 3.9 Genes related to amino acid transporters were regulated through the Tor1-Lkh1 pathway

**Figure 8:**
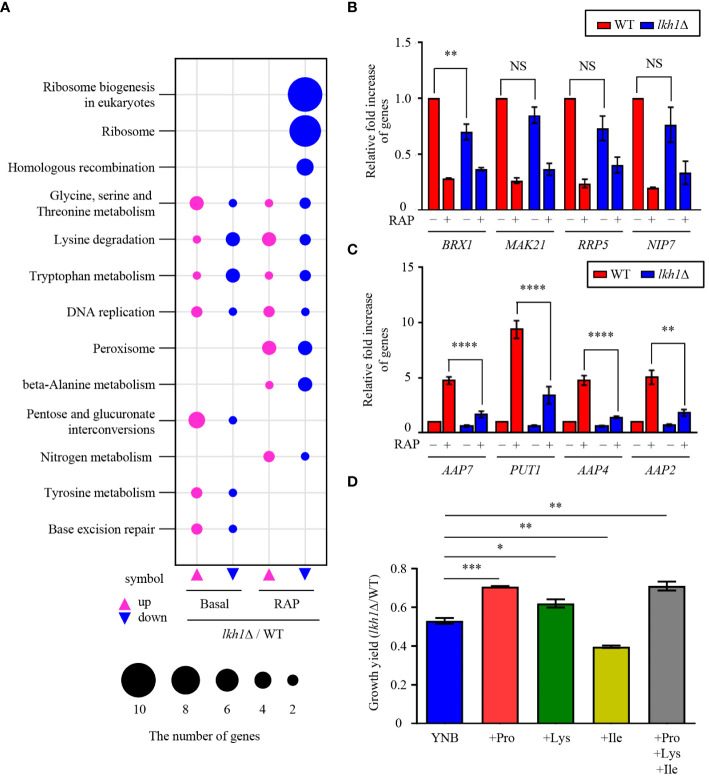
Transcriptome analysis of *lkh1*Δ mutant in response to rapamycin. **(A)** Significant differences in KEGG pathway analysis were observed between basal and rapamycin-treatment conditions (3 ng/mL) in *lkh1*Δ mutants. **(B, C)** Expression levels of genes encoding genes related to ribosome synthesis, small nuclear RNA, and amino acid transporter were verified by qRT-PCR analysis using cDNA of WT and *lkh1*Δ strains with or without rapamycin treatment (3 ng/mL). Three independent biological experiments with duplicate technical replicates were performed. Error bars indicate S.E.M. Statistical significances of the differences were determined by one-way ANOVA with Bonferroni’s multiple-comparison test (NS: not significant; ** *P* < 0.01; **** *P* < 0.0001). **(D)** Growth yield was calculated by measuring the growth curve of each strain. Each strain was grown in a minimal medium containing certain nitrogen sources (2 mM proline, 2 mM isoleucine, 2 mM lysine, and all together) Error bars indicate S.E.M. Statistical significances of the differences were determined by one-way ANOVA with Bonferroni’s multiple-comparison test. (* *P* < 0.05; ** *P* < 0.01; *** *P* < 0.001).

In this study, we observed that Tor1 acts as an upstream regulator of Lkh1. Our previous study reported that Tor1 regulates the expression of genes involved in transmembrane transport, ribosome synthesis, and metabolic processes (So et al., 2019). To elucidate the signaling circuitry downstream of CnLkh1 in the TOR1-Lkh1 pathway, we conducted RNA-seq analysis comparing the transcriptome profiles of the WT strain and the *lkh1*Δ mutants with or without rapamycin treatment. Under the basal condition, the expression levels of 409 genes in the *lkh1*Δ mutant were either increased (276 genes) or decreased (133 genes) compared to WT in a statistically significant manner (*P* < 0.05, | log2 (fold change) | >= 2) (**Supplementary Figure S4A** and **Supplementary Table S3**). Under rapamycin treatment, the expression levels of 452 genes in *lkh1*Δ mutant were either increased (290 genes) or decreased (162 genes) compared to WT in a statistically significant manner (*P* < 0.05, | log2 (fold change) | >= 2) (**Supplementary Figure S4B** and **Supplementary Table S3**). Based on the KEGG pathway analysis, we observed that CnLkh1 was involved in amino acid metabolism, DNA replication, and base excision repair in the absence of rapamycin treatment. Under rapamycin treatment, CnLkh1 was observed to be involved in ribosomal biogenesis (**Figure 8A**). To determine whether Lkh1 regulates the expression of genes involved in ribosome synthesis and small nuclear RNA processing [CNAG_02382 (*BRX1*), CNAG_04365 (*MAK21*), CNAG_02437 (*RRP5*), CNAG_04072 (*NIP7*), CNAG_06318 (*YTM1*), and CNAG_06535 (*UTP30*)], qRT-PCR analysis was performed. Notably, only *BRX1* demonstrated lower expression in the *lkh1*Δ mutant compared to that in WT. Conversely, the *MAK21*, *RRP5*, *NIP7*, *YTM1*, and *UTP30* expression levels demonstrated no significant differences between WT and *lkh1*Δ mutants (**Figure 8B** and **Supplementary Figure S4C**). The transcriptome analysis showed that expression levels of amino acid transporter or amino acid metabolism genes [CNAG_02049 (*PUT1*), CNAG_07902 (*AAP2*), CNAG_05345 (*AAP7*), and CNAG_00597 (*AAP4*)] were reduced in the *lkh1*Δ mutant. We observed that the *PUT1*, *AAP2*, *AAP4*, and *AAP7* expression levels were similar between WT and *lkh1*Δ mutant under basal conditions. However, the induction levels in the *lkh1*Δ mutant were lower than those in WT (**Figure 8C**). Put1 is involved in proline production (Lee et al., 2011). Aap4 and Aap2 are required for transporting lysine and isoleucine, respectively, as the sole nitrogen source (Martho et al., 2016). To determine whether Lkh1 is required for cell growth when a single amino acid serves as the sole nitrogen source, we monitored the growth of each strain in a minimal medium supplemented with various nitrogen sources, including lysine, isoleucine, and proline. We observed that the growth of the *lkh1*Δ mutant in YNB including proline or lysine, but not isoleucine, was partially restored compared to that of the *lkh1*Δ mutant in the YNB medium alone (**Figure 8D**). However, the growth of the *lkh1*Δ mutant in YNB medium supplemented with all amino acids (proline, isoleucine, and lysine) was similar to those in YNB supplemented with either proline (**Figure 8D**). Collectively, Lkh1 is required for proline- and lysine-dependent growth of *C*. *neoformans*.

### 3.10 Lkh1 positively regulated the virulence factor formation

**Figure 9:**
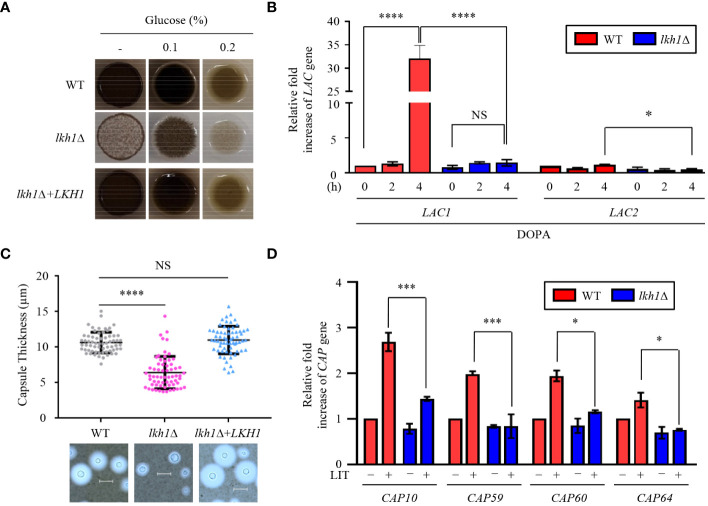
Lkh1 was involved in capsule and melanin production of *C. neoformans*. **(A)** WT (H99), *lkh1*Δ (KW1451), and *lkh1*Δ+*LKH1* (KW1633) strains were spotted on L-DOPA medium (0.1%, 0.2% glucose, and without glucose) at 30°C for 3 days. **(B, D)** Expression levels of laccase genes (*LAC1* and *LAC2*) and capsule-related genes (*CAP10*, *CAP59*, *CAP60*, and *CAP64*) were verified by qRT-PCR analysis using cDNA of WT and *lkh1*Δ mutant. Three independent biological experiments with duplicate technical replicates were performed. Error bars indicate S.E.M. Statistical significances of the differences were determined by one-way ANOVA with Bonferroni’s multiple-comparison test. (* *P* < 0.05; *** *P* < 0.001; **** *P* < 0.0001; NS: not significant) **(C)** The WT, *lkh1*Δ, and *lkh1*Δ+*LKH1* strains were cultured on a Littman medium for capsule production at 30°C for 3 days. The scale bar indicates 10 μm.

*C. neoformans* possesses several virulence factors for survival and proliferation within the host. One such factor is melanin, a dark brown polyphenolic pigment with antiphagocytic and antioxidant properties (Eisenman and Casadevall, 2012). To investigate the role of Lkh1 in melanin production, we assessed the melanin production levels of the *lhk1*Δ mutant on the _L_-DOPA medium. We observed that the *lkh1*Δ mutant produced less melanin compared to WT and its complemented strain (**Figure 9A**). In high glucose concentration conditions, which is a melanin-repressed condition (Jin et al., 2020), the difference in melanin production between *lkh1*Δ and WT strains was obvious. Despite a long incubation period (7 days), melanin production in *lkh1*Δ mutant did not recover to WT level (Data not shown). Given that the *lkh1*Δ mutant exhibited growth defect, we wondered whether the reduced melanin production in the *lkh1*Δ mutant originated from its growth defect or the influence on melanin-producing genes encoding laccases (*LAC1* and *LAC2*). To test this hypothesis, we measured the *LAC1* and *LAC2* expression levels under melanin-induced conditions. We observed that the induction levels of *LAC1* in the *lkh1*Δ mutant were significantly lower compared to the WT, and the *LAC2* expression level in the *lkh1*Δ mutant was slightly lower than that in WT (**Figure 9B**).

The polysaccharide capsule, located outside the fungal cell wall and enabling the fungus to evade immune recognition and destruction (Chen et al., 2022), is another major virulence factor of *C. neoformans*. Next, we compared capsule production between WT and *lkh1*Δ mutants. Similar to the melanin production, *lkh1*Δ mutant showed reduced capsule production compared to WT and complemented strains (**Figure 9C**). We wondered whether the decrease in capsule production in the *lkh1*Δ mutant resulted from its growth defect or differences in gene expression related to capsule production. We observed that induction levels of capsule production-related genes including *CAP10*, *CAP59*, *CAP60*, and *CAP64* in the *lkh1*Δ mutant were significantly lower compared to those in WT under capsule induction conditions (**Figure 9D**). To determine whether the kinase activity of Lkh1 influences the formation of virulence factors, we performed melanin and capsule production assays. Strikingly, the *LKH1^D546A^* strains produced less melanin and capsule production similar to the *lkh1*Δ mutant (**Supplementary Figure S5**). Taken together, these findings indicate that Lkh1 is involved in the formation of both capsule and melanin in *C. neoformans*.

### 3.11 Lkh1 was required for virulence in *C. neoformans*

**Figure 10:**
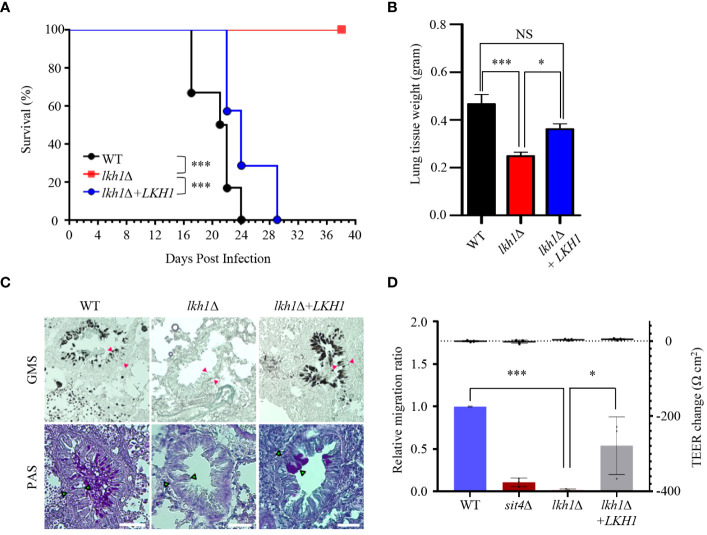
Lkh1 was essential for the virulence of *C neoformans*. **(A)** *C neoformans* WT (H99), *lkh1*Δ (KW1451), and *lkh1*Δ+*LKH1* (KW1633) strains were nasally inoculated into the 7-week-old female Balb/c mice and monitored. Survival curve of infected mice (n=7). **(B)** Mice were sacrificed for histochemistry at 16 dpi (n=5). **(B)** Comparison of lung tissue weight measurements. **(C)** Grocott’s methenamine silver (GMS) and Periodic Acid-Schiff (PAS) staining of lung tissues of mice sacrificed 16 days after infection. Red arrows indicate the walls of small bronchioles. Scale bar means 0.04 mm. **(D)** BBB translocation assays using hCMEC/D3-coated transwell. Plates with an inoculum of 10^5^ cells of WT, *lkh1*Δ, *lkh1* Δ+*LKH1*, and *sit4*Δ strains were incubated at 37°C in a CO_2_ incubator for 24 h, and the number of yeast cells passing through the hCMEC/D3-coated transwell was measured by CFU. The *sit4*Δ mutant was used as a negative control. The left and right Y axes indicate the migrated CFU and the trans-endothelial electrical resistance (TEER) value, respectively. Three independent biological experiments with duplicate technical replicates were performed. Error bars indicate S.E.M. Statistical significances of the differences were determined by one-way ANOVA with Bonferroni’s multiple-comparison test. (* *P* < 0.05; *** *P* < 0.001).

Our experiment results, which showed that the *lkh1*Δ mutant displayed a growth defect at 37°C, along with reduced melanin and capsule productions, indicate that it plays an essential role in virulence. To demonstrate this hypothesis, we assessed the virulence of the *lkh1*Δ mutant in a murine model of cryptococcosis. We found that the *lkh1*Δ mutant was completely avirulent. Although the mice infected with *lkh1*Δ+*LKH1* showed delayed symptoms compared to mice infected with the WT, all the mice were symptomatic within 30 days post-infection (**Figure 10A**). To investigate the effect of Lkh1 on pulmonary growth, we observed the lung tissues of infected mice at 16 days post-infection. Lung tissues were much heavier in WT and *lkh1*Δ+*LKH1* strain-infected mice compared to *lkh1*Δ mutant-infected mice (**Figure 10B**). In particular, no evidence of fungal infection was observed around the bronchioles of *lkh1*Δ mutant-infected lung (**Figure 10C**, upper panel for fungal organisms; **Figure 10C**, lower panel for immune cell clusters). To further demonstrate that Lkh1 is required for BBB-traversal activity, we performed an *in vitro* BBB transmigration assay. Expectedly, BBB transmigration of the *lkh1*Δ mutant was much lower than that of the WT or *lkh1*Δ+*LKH1* strains (**Figure 10D**). These results indicate that Lkh1 plays a crucial role in the virulence of *C. neoformans* by governing its BBB crossing capacity.

## 4 Discussion

**Figure 11:**
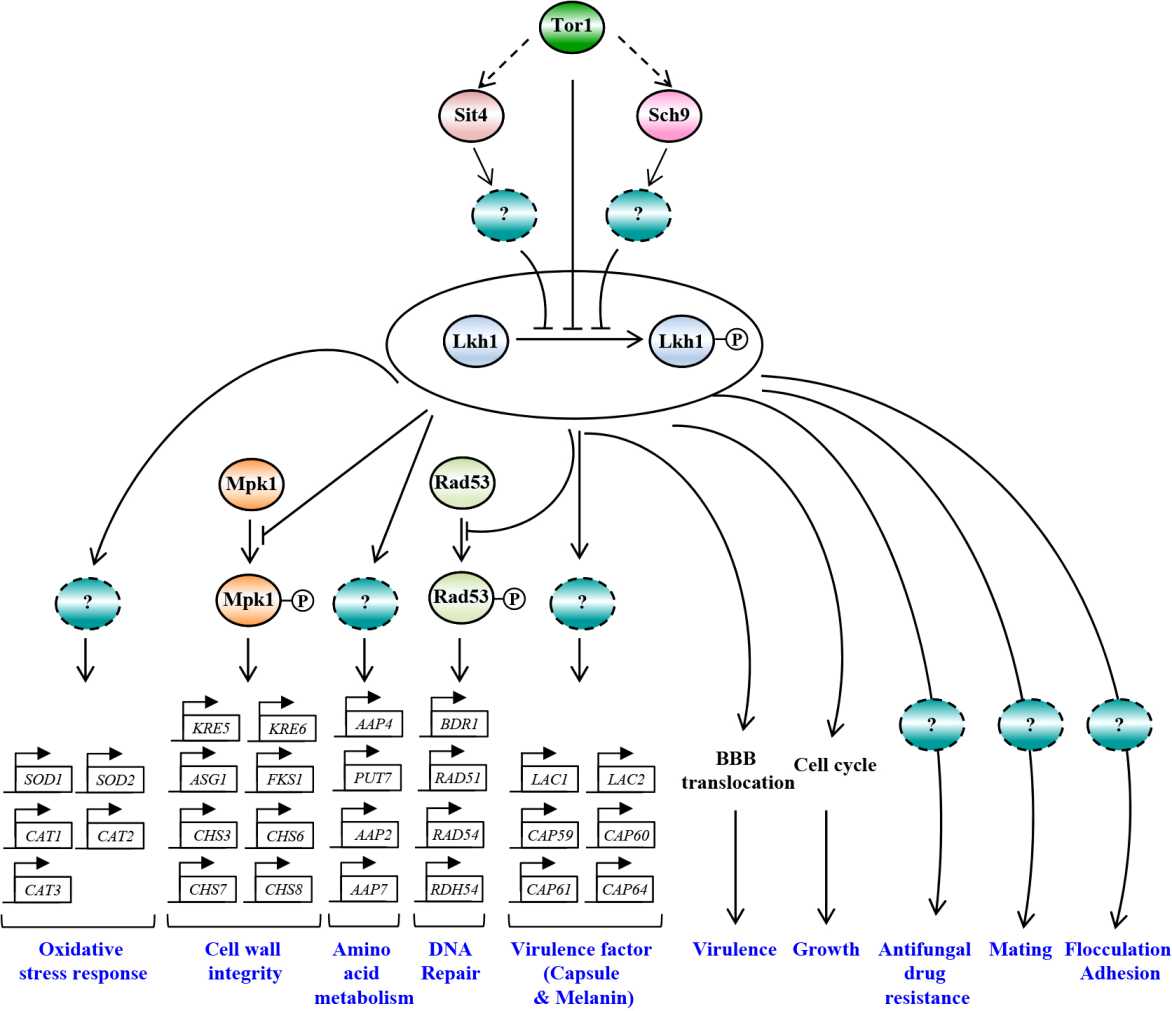
The proposed model of Lkh1-dependent pathway in *C. neoformans*. In response to extraneous stress stimuli, Lkh1 orchestrates a myriad of stress responses including oxidative stress response, preservation of cell wall integrity, DNA damage response, and modulation of amino acid metabolism via the regulation of downstream target gene expression. Additionally, Lkh1 contributes to antifungal drug resistance, mating, flocculation, adhesion, and intrinsic growth. Notably, Lkh1 plays a pivotal role in virulence by modulating the translocalization of the blood-brain barrier (BBB).

In this study, we demonstrated the pleiotropic roles of CnLkh1 in diverse stress responses, sexual differentiation, and antifungal drug resistance. Furthermore, we characterized its downstream regulatory networks governed by CnLkh1 using transcriptome analysis and confirmed that Lkh1 acts as one of the downstream kinases in the TOR signaling pathway (**Figure 11**).

Although *Cryptococcus LKH1* deletion caused defects in growth and stress responses akin to other fungal species lacking the *LKH1* homologous gene, its regulatory mechanisms appeared to be distinct among fungi. In *U. maydis*, Lkh1 regulates the DNA damage response in a homologous recombination (HR) pathway-independent manner (de Sena-Tomás et al., 2015). *M. oryzae* Kns1 controls the expression of genes involved in DNA damage checkpoints and cell cycle regulation (Li et al., 2022). Our study reported that CnLkh1 regulates DNA damage response in both Rad53-dependent and -independent manners. Second, in *S. cerevisiae*, both catalytic and non-catalytic activities of LAMMER kinase are required for the stress response (Lee et al., 2012). MoKns1 physically interacts with Atg18 via a non-kinase domain (Li et al., 2022). Furthermore, the strain with a mutation at Q488 in the kinase domain of Lkh1 showed greater susceptibility to DNA damage stress than the strain lacking the entire *LKH1* gene in *U. maydis* (de Sena-Tomás et al., 2015). However, the catalytic activity of Lkh1 in *C. neoformans* is mainly responsible for most stress responses and virulence factor formation, except for fluconazole resistance. Third, our study revealed that Lkh1 phosphorylation was dependent on the TOR signaling pathway. A unique finding was that *Cryptococcus* Lkh1 was already phosphorylated in the absence of external stress. In this context, numerous genes were intrinsically up-regulated or down-regulated in the *lkh1*Δ mutant compared to WT at basal condition. These data indicate that Lkh1 in *C. neoformans* intrinsically contributes to diverse cellular responses regardless of external stress.

Fungal cell walls serve multifaceted roles in host interaction and are indispensable for maintaining cellular integrity. Integral constituents within the cell wall are recognized as major fungal pathogen-associated molecular patterns (PAMPs). The perturbation in cell composition resulting from the deletion of *LKH1* influences PAMPs, thereby diminishing virulence in *A. fumigatus* (Lim et al., 2021). In *C. albicans*, genes responsible for encoding GPI-anchored proteins exhibit substantial upregulation in the *kns1*Δ mutant compared to WT (Lim et al., 2020). Our investigation also elucidated that perturbation in *LKH1* changes in chitin content. This observation is substantiated by the impact of Lkh1 on flocculation and adhesion activities in *C. neoformans*. Consequently, modifications in the cell wall components of the *lkh1*Δ mutant may contribute to the attenuation of virulence in *C. neoformans*.

In this study, we made a notable observation that ergosterol contents in the *lkh1*Δ mutant were similar to that in WT, although the *lkh1*Δ mutant exhibited opposite resistance to amphotericin B and fluconazole, respectively. This result contrasts with strains showing the opposite resistance to polyene and azole drugs, which typically contain more or less ergosterol contents in yeast (Young et al., 2003; Ko et al., 2009). However, susceptibility or resistance to azole/polyene drugs does not depend on the ergosterol content alone. Supporting this, no significant correlation was observed between azole/polyene susceptibility patterns and ergosterol content in clinical yeast strains such as *C. albicans* and *C. neoformans* (Gomez-Lopez et al., 2011). Given that antifungal drugs penetrate the cell wall before interacting with the cell membrane, changes in cell wall constituents may contribute to susceptibility to polyene or azole drugs. Remarkably, reduced chitin content in the cell wall has been shown to decrease the susceptibility to amphotericin B in *Kluyveromyces* and *Candida* strains (Bahmed et al., 2003). Similarly, increased chitin levels were observed in fluconazole-resistant *Candida auris* clinical isolates (Shahi et al., 2022). There, alteration in cell wall constituents of the *lkh1*Δ mutant might induce polyene or azole drug susceptibility.

Our finding that Lkh1 is essential for virulence reinforces the pivotal role of the TOR signaling pathway in the pathogenicity of *C. neoformans*. Our previous studies revealed that TOR-dependent cellular processes such as ribosome genesis, carbon utilization, and amino acid metabolism are required for BBB infection and survival in the brain (Lee et al., 2020). Supporting this notion, Sch9 kinase and Sit4 phosphatase have been identified as necessary components for BBB infection (Jin et al., 2020). Recently, the TOR pathway has been implicated in adaptation to the host environment through Ypk1-dependent phospholipid asymmetry remodeling (Ristow et al., 2023). Besides kinases and phosphatases, Zfp1, which is homologous to Sfp1 and is involved in nutrient sensing and the cell cycle as a downstream transcription factor of TOR signaling in *S. cerevisiae*, is required for virulence in *C. neoformans*. Especially, the *zfp1*Δ mutant shows a reduced fungal burden in the brain and spleen (Fan et al., 2019). A previous study reported that TF mutants (*hlh5*Δ, *bzp1*Δ, *hob1*Δ, *ada2*Δ, *crz1*Δ, *skn7*Δ, and *atf1*Δ mutants) exhibit susceptibility or resistance in response to rapamycin and some of them display reduced virulence (Jung et al., 2015; So et al., 2019). Therefore, the TOR pathway appears essential for virulence in *C. neoformans*.

In addition to the upstream regulators of LAMMER kinases, the genome-wide downstream genes regulated by LAMMER kinases in fungal pathogens remain elusive. To the best of our knowledge, this is the first transcriptome analysis of a LAMMER kinase mutant in a human fungal pathogen. The transcriptome analyses in this study provide insights into *LKH1* deletion-induced phenotypic changes in response to diverse stress responses and sexual differentiation. First, among Lkh1-dependent genes, expression levels of nucleotide metabolism-related genes were changed. *YNK1*, which encodes a nucleoside diphosphate kinase, catalyzes the transfer of phosphates from nucleoside triphosphates and is induced during DNA replication stress (Yang et al., 2009). Likewise, *HAM1*, which encodes nucleoside triphosphate pyrophosphohydrolase, is involved in non-canonical purine and pyrimidine metabolism, and its protein level increases in response to DNA replication stress (Takayama et al., 2007). Our previous studies reveal that *Cryptococcus YNK1* appears to be essential for growth, while the *ham1*Δ mutant showed WT-levels of resistance in response to stress responses (Jin et al., 2020). Supporting this, nucleotide metabolism is crucial for DNA damage stress as well as DNA replication stress (Jung et al., 2022). These data indicate that *Cryptococcus* Lkh1 may function in response to DNA damage stress by regulating nucleotide metabolism. Secondly, nitrogen metabolism has been characterized by its role in virulence, stress response, autophagy, sexual differentiation, and capsule and melanin production in *C. neoformans* (Kmetzsch et al., 2011; Lee et al., 2011; Frazzitta et al., 2013; Jiang et al., 2020). Although the TOR signaling pathway controls nitrogen metabolism in *S. cerevisiae* (Crespo et al., 2002), the regulatory mechanism of TOR-mediated nitrogen metabolism has not been elucidated in *C. neoformans*. Our transcriptome data provided evidence that Lkh1 controls nitrogen metabolism by regulating the expression of amino acid transporter genes. Supporting this, the phenomenon that *lkh1*Δ mutant showed reduced mating efficiency, capsule production, and melanin biosynthesis, is similar to the phenotype observed in the strains deleted with genes related to amino acid metabolism (Kmetzsch et al., 2011; Lee et al., 2011). However, in this state, we need to identify TOR-dependent transcription factors that regulate nitrogen metabolism and further elucidate the relationship between these transcription factors and Lkh1 in *C. neoformans*.

Although LAMMER kinases in fungi are evolutionarily conserved and play pleiotropic roles in stress responses and differentiation, information about crosstalks between LAMMER kinase and other signaling pathways is limited. However, our study revealed that Lkh1 is partially interconnected with the Mpk1 pathway for cell wall stress response and the Rad53-Bdr1 pathway for the DNA damage response. In yeast, crosstalk between TOR and cAMP/PKA pathway has been partially elucidated. For instance, activation signals of Rim15, a protein kinase involved in cell proliferation in response to nutrient, are integrated from the TOR and PKA pathways (Pedruzzi et al., 2003). Furthermore, the constitutive activation of the RAS/cAMP pathway inhibits rapamycin-induced responses (Schmelzle et al., 2004). Defects in the capsule and melanin productions in *lkh1*Δ mutant were also observed, akin to when the PKA/cAMP pathway is impaired. Therefore, LAMMER kinase, Lkh1 may have crosstalk with the cAMP/PKA pathway as the downstream factor of the TOR pathway in *C. neoformans* and further research will be necessary to address this relationship in the future.

## Data availability statement

The datasets presented in this study can be found in online repositories. The names of the repository/repositories and accession number(s) can be found below: https://www.ncbi.nlm.nih.gov/geo/, GSE233612.

## Ethics statement

The animal study was approved by Korea Zoonosis Research Institute. The study was conducted in accordance with the local legislation and institutional requirements.

## Author contributions

SK: Validation, Supervision, Writing – review & editing, Writing – original draft, Methodology, Investigation, Formal analysis, Data curation. YC: Validation, Writing – review & editing, Writing – original draft, Methodology, Investigation, Data curation. E-SK: Writing – review & editing, Writing – original draft, Methodology, Investigation, Formal analysis. K-TL: Writing – review & editing, Writing – original draft, Supervision, Investigation. Y-SB: Writing – review & editing, Writing – original draft, Supervision, Investigation. K-WJ: Formal analysis, Writing – review & editing, Writing – original draft, Validation, Supervision, Investigation, Funding acquisition, Data curation, Conceptualization.

## Supplementary material

Supplementary Figure 1 Construction of the *lkh1*Δ, *rad53*Δ *lkh1*Δ, *mpk1*Δ *lkh1*Δ, Rad53-4×FLAG *lkh1*Δ, *LKH1*-*4×FLAG*, *LKH1-4×FLAG sit4*Δ, and *LKH1-4×FLAG sch9*Δ mutants. (A, B, H, J, L) Diagram for *LKH1* gene disruption in serotype A (*MAT*α) H99 strain, *rad53*Δ, *mpk1*Δ, Rad53-4×FLAG mutant, *MAT*a KN99, *LKH1*-*4×FLAG*, *LKH1-4×FLAG sit4*Δ, and *LKH1-4×FLAG sch9*Δ mutants. (C, D, E, F, G, I, K, and M) The correct gene disruptions were confirmed by Southern blot analysis using genomic DNAs digested with the indicated restriction enzyme.

Supplementary Figure 2 Lkh1 was involved in the cell cycle progression. **(A)** WT (H99), *lkh1*Δ (KW1451), and *lkh1*Δ+*LKH1* (KW1633) strains were grown in liquid YPD medium for 16 h at 30°C and subcultured in fresh liquid YPD medium at 37°C. Cell density was measured at an optical density of 600 nm every 2 h. **(B)** Intracellular measurement of ATP level in *lkh1*Δ mutant. WT (H99), *lkh1*Δ (KW1451), and *lkh1*Δ+*LKH1* (KW1633) strains were grown in liquid YPD medium for 16 h at 30°C and washed using PBS. The ATP contents of strains were calculated by measuring luminescence. ATP concentrations were normalized using soluble-protein concentrations for each sample. Three independent biological experiments with duplicate technical replicates were performed. Error bars indicate S.E.M. Statistical significances of the differences were determined by one-way ANOVA with Bonferroni’s multiple-comparison test. (NS: not significant) **(C)** Cell cycle analysis of *lkh1*Δ mutant via flow cytometry. Cells were stained with propidium iodine (PI). The three biological replicates were performed. **(D)** Morphological test of *lkh1*Δ mutant. Cells were fixed with ethanol and observed using differential interference contrast (DIC) (Nikon Eclipse, Japan).

Supplementary Figure 3 Spotting assay to confirm the functionality of Lkh1-4×FLAG protein. WT (H99), *lkh1*Δ (KW1451), *lkh1*Δ+*LKH1* (KW1633), and Lkh1-4×FLAG strain (KW1755) strains were cultured in liquid YPD medium overnight at 30°C, 10-fold serially diluted and spotted on YPD medium containing 2 μg/mL bleomycin, 0.8 mg/mL caffeine, 0.8 mg/mL CFW, 1.2% CR, 0.02% MMS, 0.15 μg/mL 4-NQO, 90 mM HU, 5 mM cisplatin, 1 μg/mL Amphotericin B, or 18 μg/mL fluconazole. For the UV-C resistance test, the serially-diluted cells were spotting onto the YPD medium, and the indicated dose of UV-C was exposed. The plates were further incubated at 30°C and photographed daily for 2-4 days.

Supplementary Figure 4 Volcano plot and expression of genes using data from transcriptome analysis. **(A)** Volcano plots were shown between WT and *lkh1*Δ mutant (KW1451) at basal time. Yellow dots mean genes in a statistically significant manner (log_2_ Fold Change [*lkh1*Δ/WT] >= 2 and *P* < 0.05) and blue dots mean genes in a statistically significant manner (log_2_ Fold Change [*lkh1*Δ/WT] <= -2 and *P* < 0.05). **(B)** Volcano plots were shown between WT and *lkh1*Δ mutant (KW1451)) under rapamycin (3 ng/mL) treatment. Yellow dots mean genes in a statistically significant manner (log_2_ Fold Change [*lkh1*Δ/WT] >= 2 and *P* < 0.05) and blue dots mean genes in a statistically significant manner (log_2_ Fold Change [*lkh1*Δ/WT] <= -2 and *P* < 0.05) **(C)** Expression levels of genes encoding genes related ribosome synthesis were verified by qRT-PCR analysis using cDNA of WT and *lkh1*Δ strains with or without rapamycin treatment (3 ng/mL). Three independent biological experiments with duplicate technical replicates were performed. Error bars indicate S.E.M. Statistical significances of the differences were determined by one-way ANOVA with Bonferroni’s multiple-comparison test (NS: not significant).

Supplementary Figure 5 Catalytic activity of Lkh1 was involved in the capsule and melanin production of *C. neoformans*. **(A)** The WT (H99), *lkh1*Δ (KW1451), *lkh1*Δ+*LKH1* (KW1633), and *LKH1* ^D546A^ (KW1958) strains were spotted on _L_-DOPA medium with 0.2% glucose and without glucose at 30°C and 37°C for 3 days. **(B)** The WT (H99), *lkh1*Δ (KW1451), *lkh1*Δ+*LKH1* (KW1633), and *LKH1* ^D546A^ (KW1958) strains were cultured on a Littman medium for capsule production at 30°C for 3 days. The scale bar indicates 10 μm.

## References

[B1] Arthington-Skaggs B. A. Jradi H. Desai T. Morrison C. J. (1999). Quantitation of ergosterol content: novel method for determination of fluconazole susceptibility of *Candida albicans* . J. Clin. Microbiol. 37, 3332–3337. doi: 10.1128/JCM.37.10.3332-3337.1999 10488201 PMC85559

[B2] Bahmed K. Bonaly R. Coulon J. (2003). Relation between cell wall chitin content and susceptibility to amphotericin B in *Kluyveromyces*, *Candida* and *Schizosaccharomyces* species. Res. Microbiol. 154, 215–222. doi: 10.1016/S0923-2508(03)00049-4 12706511

[B3] Banks I. R. Specht C. A. Donlin M. J. Gerik K. J. Levitz S. M. Lodge J. K. (2005). A chitin synthase and its regulator protein are critical for chitosan production and growth of the fungal pathogen *Cryptococcus neoformans* . Eukaryot. Cell 4, 1902–1912. doi: 10.1128/EC.4.11.1902-1912.2005 16278457 PMC1287864

[B4] Bender J. Fink G. R. (1994). Afc1, A LAMMER kinase from *Arabidopsis thaliana*, activates ste12-dependent processes in yeast. Proc. Natl. Acad. Sci. U. S. A., 12105–12109, 91. doi: 10.1073/pnas.91.25.12105 7991592 PMC45385

[B5] Chang M. Sionov E. Khanal Lamichhane A. Kwon-Chung K. J. Chang Y. C. (2018). Roles of three *Cryptococcus neoformans* and *Cryptococcus gattii* efflux pump-coding genes in response to drug treatment. Antimicrob. Agents Chemother. 62, 10.1128/Aac. 01751–17. doi: 10.1128/AAC.01751-17 PMC5913978 29378705

[B6] Chen Y. Shi Z. W. Strickland A. B. Shi M. (2022). *Cryptococcus neoformans* infection in the central nervous system: the battle between host and pathogen. J. Fungi 8, 1069. doi: 10.3390/jof8101069 PMC9605252 36294634

[B7] Cho S. J. Kim Y. H. Park H. M. Shin K. S. (2010). Possible roles of LAMMER kinase Lkh1 in fission yeast by comparative proteome analysis. Mycobiology 38, 108–112. doi: 10.4489/MYCO.2010.38.2.108 23956636 PMC3741559

[B8] Choi Y. K. Kang E.-H. Park H.-M. (2014). Role of lammer kinase in cell wall biogenesis during vegetative growth of *Aspergillus nidulans* . Mycobiology 42, 422–426. doi: 10.5941/MYCO.2014.42.4.422 25606019 PMC4298851

[B9] Choi Y. Yu S.-R. Lee Y. Na A.-Y. Lee S. Heitman J.. (2024). Casein kinase 2 complex: A central regulator of multiple pathobiological signaling pathways in *Cryptococcus neoformans* . Mbio 15, E03275–E03223. doi: 10.1128/mbio.03275-23 38193728 PMC10865844

[B10] Cramer K. L. Gerrald Q. D. Nichols C. B. Price M. S. Alspaugh J. A. (2006). Transcription factor nrg1 mediates capsule formation, stress response, and pathogenesis in *Cryptococcus neoformans* . Eukaryot. Cell 5, 1147–1156. doi: 10.1128/EC.00145-06 16835458 PMC1489281

[B11] Crespo J. L. Powers T. Fowler B. Hall M. N. (2002). The TOR-controlled transcription activators Gln3, Rtg1, and Rtg are regulated in response to intracellular levels of glutamine. Proc. Natl. Acad. Sci. U. S. A. 99, 6784–6789. doi: 10.1073/pnas.102687599 11997479 PMC124480

[B12] de Sena-Tomás C. Sutherland J. H. Milisavljevic M. Nikolic D. B. Përez-Martïn J. Kojic M.. (2015). Lammer kinase contributes to genome stability in *Ustilago maydis* . DNA Repair 33, 70–77. doi: 10.1016/j.dnarep.2015.05.011 26176563 PMC4526389

[B13] Di Como C. J. Arndt K. T. (1996). Nutrients, via the TOR proteins, stimulate the association of Tap42 with type 2a phosphatases. Genes. Dev. 10, 1904–1916. doi: 10.1101/gad.10.15.1904 8756348

[B14] Duclos B. Marcandier S. Cozzone A. J. (1991). Chemical properties and separation of phosphoamino acids by thin-layer chromatography and/or electrophoresis. Methods Enzymol. 201, 10–21. doi: 10.1016/0076-6879(91)01004-L 1943759

[B15] Eisenman H. C. Casadevall A. (2012). Synthesis and assembly of fungal melanin. Appl. Microbiol. Biotechnol. 93, 931–940. doi: 10.1007/s00253-011-3777-2 22173481 PMC4318813

[B16] Ellis D. H. Pfeiffer T. J. (1990). Ecology, life cycle, and infectious propagule of *Cryptococcus neoformans* . Lancet 336, 923–925. doi: 10.1016/0140-6736(90)92283-N 1976940

[B17] Fan C.-L. Han L.-T. Jiang S.-T. Chang A.-N. Zhou Z.-Y. Liu T.-B. (2019). The Cys2His2 zinc finger protein Zfp1 regulates sexual reproduction and virulence in *Cryptococcus neoformans* . Fungal. Genet. Biol. 124, 59–72. doi: 10.1016/j.fgb.2019.01.002 30630094

[B18] Fan Y. Lin X. (2018). Multiple applications of A transient Cryptococcus coupled with electroporation (Trace) system in the *Cryptococcus neoformans* species complex. Genetics 208, 1357–1372. doi: 10.1534/genetics.117.300656 29444806 PMC5887135

[B19] Frazzitta A. E. Vora H. Price M. S. Tenor J. L. Betancourt-Quiroz M. Toffaletti D. L.. (2013). Nitrogen source-dependent capsule induction in human-pathogenic *Cryptococcus* species. Eukaryot. Cell 12, 1439–1450. doi: 10.1128/EC.00169-13 23975889 PMC3837930

[B20] Garcia J. Shea J. Alvarez-Vasquez F. Qureshi A. Luberto C. Voit E. O.. (2008). Mathematical modeling of pathogenicity of *Cryptococcus neoformans* . Mol. Syst. Biol. 4, 183. doi: 10.1038/msb.2008.17 18414484 PMC2387229

[B21] Gilbert N. M. Donlin M. J. Gerik K. J. Specht C. A. Djordjevic J. T. Wilson C. F.. (2010). Kre genes are required for β-1, 6-glucan synthesis, maintenance of capsule architecture and cell wall protein anchoring in *Cryptococcus neoformans* . Mol. Microbiol. 76, 517–534. doi: 10.1111/j.1365-2958.2010.07119.x 20384682 PMC2969852

[B22] Gomez-Lopez A. Buitrago M. J. Rodriguez-Tudela J. L. Cuenca-Estrella M. (2011). *In vitro* antifungal susceptibility pattern and ergosterol content in clinical yeast strains. Rev. Iberoam. Micol. 28, 100–103. doi: 10.1016/j.riam.2010.12.003 21251996

[B23] Gottfredsson M. Perfect J. R. (2000). Fungal meningitis. Semin. Neurol., 20, 307–322. doi: 10.1055/s-2000-9394 11051295

[B24] Hanes J. Von Der Kammer H. Klaudiny J. Scheit K. H. (1994). Characterization by cDNA cloning of two new human protein kinases. Evidence by sequence comparison of A new family of mammalian protein kinases. J. Mol. Biol. 244, 665–672. doi: 10.1006/jmbi.1994.1763 7990150

[B25] Idnurm A. Bahn Y. S. Nielsen K. Lin X. Fraser J. A. Heitman J. (2005). Deciphering the model pathogenic fungus *Cryptococcus neoformans* . Nat. Rev. Microbiol. 3, 753–764. doi: 10.1038/nrmicro1245 16132036

[B26] Jang E-H Kim J-S Yu S-R Bahn Y-S (2022). Unraveling Capsule Biosynthesis and Signaling Networks in *Cryptococcus neoformans* . Microbiol. Spectr. 10, e02866–22. doi: 10.1128/spectrum.02866-22 36287085 PMC9769619

[B27] Jiang N. Benard C. Y. Kebir H. Shoubridge E. A. Hekimi S. (2003). Human clk2 links cell cycle progression, apoptosis, and telomere length regulation. J. Biol. Chem. 278, 21678–21684. doi: 10.1074/jbc.M300286200 12670948

[B28] Jiang S. T. Chang A. N. Han L. T. Guo J. S. Li Y. H. Liu T. B. (2020). Autophagy regulates fungal virulence and sexual reproduction in *Cryptococcus neoformans* . Front. Cell. Dev. Biol. 8, 374. doi: 10.3389/fcell.2020.00374 32528953 PMC7262457

[B29] Jin J.-H. Lee K.-T. Hong J. Lee D. Jang E.-H. Kim J.-Y.. (2020). Genome-wide functional analysis of phosphatases in the pathogenic fungus *Cryptococcus neoformans* . Nat. Commun. 11, 4212. doi: 10.1038/s41467-020-18028-0 32839469 PMC7445287

[B30] Joseph-Horne T. Hollomon D. Loeffler R. Kelly S. L. (1995). Cross-resistance to polyene and azole drugs in *Cryptococcus neoformans* . Antimicrob. Agents. Chemother. 39, 1526–1529. doi: 10.1128/AAC.39.7.1526 7492098 PMC162775

[B31] Jung K.-W. Jung J.-H. Park H.-Y. (2021). Functional roles of homologous recombination and non-homologous end joining in DNA damage response and microevolution in *Cryptococcus neoformans* . J. Fungi 7, 566. doi: 10.3390/jof7070566 PMC8307084 34356945

[B32] Jung K.-W. Kwon S. Jung J.-H. Bahn Y.-S. (2022). Essential roles of ribonucleotide reductases under DNA damage and replication stresses in *Cryptococcus neoformans* . Microbiol. Spectr. 10, E01044–E01022. doi: 10.1128/spectrum.01044-22 35736239 PMC9431586

[B33] Jung K.-W. Lee Y. Huh E. Y. Lee S. C. Lim S. Bahn Y.-S. (2019). Rad53-and Chk1-dependent DNA damage response pathways cooperatively promote fungal pathogenesis and modulate antifungal drug susceptibility. Mbio 10, E01726–E01718. doi: 10.1128/mBio.01726-18 30602579 PMC6315099

[B34] Jung K. W. Lee K. T. So Y. S. Bahn Y. S. (2018). Genetic manipulation of *Cryptococcus neoformans* . Curr. Protoc. Microbiol. 50, E59. doi: 10.1002/cpmc.59 30016567

[B35] Jung K.-W. Strain A. K. Nielsen K. Jung K.-H. Bahn Y.-S. (2012). Two cation transporters ena1 and nha1 cooperatively modulate ion homeostasis, antifungal drug resistance, and virulence of *Cryptococcus neoformans* via the hog pathway. Fungal. Genet. Biol. 49, 332–345. doi: 10.1016/j.fgb.2012.02.001 22343280 PMC3319253

[B36] Jung K.-W. Yang D.-H. Kim M.-K. Seo H. S. Lim S. Bahn Y.-S. (2016). Unraveling fungal radiation resistance regulatory networks through the genome-wide transcriptome and genetic analyses of *Cryptococcus neoformans* . Mbio 7, E01483–E01416. doi: 10.1128/mBio.01483-16 27899501 PMC5137497

[B37] Jung K.-W. Yang D.-H. Maeng S. Lee K.-T. So Y.-S. Hong J.. (2015). Systematic functional profiling of transcription factor networks in *Cryptococcus neoformans* . Nat. Commun. 6, 1–14. doi: 10.1038/ncomms7757 PMC4391232 25849373

[B38] Kang E.-H. Kim J.-A. Oh H.-W. Park H.-M. (2013). Lammer kinase LkhA plays multiple roles in the vegetative growth and asexual and sexual development of *Aspergillus nidulans* . PloS One 8, E58762. doi: 10.1371/journal.pone.0058762 23516554 PMC3596290

[B39] Kang W.-H. Park Y.-H. Park H.-M. (2010). The lammer kinase homolog, Lkh1, regulates tup transcriptional repressors through phosphorylation in *Schizosaccharomyces pombe* . J. Biol. Chem. 285, 13797–13806. doi: 10.1074/jbc.M110.113555 20200159 PMC2859543

[B40] Kim K.-H. Cho Y.-M. Kang W.-H. Kim J.-H. Byun K.-H. Park Y.-D.. (2001). Negative regulation of filamentous growth and flocculation by Lkh1, A fission yeast lammer kinase homolog. Biochem. Biophys. Res. Commun. 289, 1237–1242. doi: 10.1006/bbrc.2001.6128 11741326

[B41] Kim J.-S. Lee K.-T. Lee M. H. Cheong E. Bahn Y.-S. (2021). Adenylyl cyclase and protein kinase A play redundant and distinct roles in growth, differentiation, antifungal drug resistance, and pathogenicity of *Candida auris* . Mbio 12. doi: 10.1128/mBio.02729-21 PMC8524339 34663094

[B42] Kmetzsch L. Staats C. C. Simon E. Fonseca F. L. Oliveira D. L. Joffe L. S.. (2011). The gata-type transcriptional activator gat1 regulates nitrogen uptake and metabolism in the human pathogen *Cryptococcus neoformans* . Fungal Genet. Biol. 48, 192–199. doi: 10.1016/j.fgb.2010.07.011 20673806

[B43] Ko Y.-J. Yu Y. M. Kim G.-B. Lee G.-W. Maeng P. J. Kim S.. (2009). Remodeling of global transcription patterns of *Cryptococcus neoformans* genes mediated by the stress-activated HOG signaling pathways. Eukaryot. Cell 8, 1197–1217. doi: 10.1128/EC.00120-09 19542307 PMC2725552

[B44] Kraus P. R. Fox D. S. Cox G. M. Heitman J. (2003). The *Cryptococcus neoformans* MAP kinase Mpk1 regulates cell integrity in response to antifungal drugs and loss of calcineurin function. Mol. Microbiol. 48, 1377–1387. doi: 10.1046/j.1365-2958.2003.03508.x 12787363 PMC1635492

[B45] Kwon-Chung K. Sorrell T. Dromer F. Fung E. Levitz S. (2000). Cryptococcosis: clinical and biological aspects. Med. Mycol. 38, 205–213. doi: 10.1080/mmy.38.s1.205.213 11204147

[B46] Lee K.-T. Byun H.-J. Jung K.-W. Hong J. Cheong E. Bahn Y.-S. (2014). Distinct and redundant roles of protein tyrosine phosphatases Ptp1 and Ptp2 in governing the differentiation and pathogenicity of *Cryptococcus neoformans* . Eukaryot. Cell. 13, 796–812. doi: 10.1128/EC.00069-14 24728196 PMC4054275

[B47] Lee I. R. Chow E. W. Morrow C. A. Djordjevic J. T. Fraser J. A. (2011). Nitrogen metabolite repression of metabolism and virulence in the human fungal pathogen *Cryptococcus neoformans* . Genetics 188, 309–323. doi: 10.1534/genetics.111.128538 21441208 PMC3122321

[B48] Lee K. Du C. Horn M. Rabinow L. (1996). Activity and autophosphorylation of lammer protein kinases. J. Biol. Chem. 271, 27299–27303. doi: 10.1074/jbc.271.44.27299 8910305

[B49] Lee K.-T. Hong J. Lee D.-G. Lee M. Cha S. Lim Y.-G.. (2020). Fungal kinases and transcription factors regulating brain infection in *Cryptococcus neoformans* . Nat. Commun. 11, 1521. doi: 10.1038/s41467-020-15329-2 32251295 PMC7090016

[B50] Lee D. Jang E.-H. Lee M. Kim S.-W. Lee Y. Lee K.-T.. (2019). Unraveling melanin biosynthesis and signaling networks in *Cryptococcus neoformans* . Mbio 10. doi: 10.1128/mBio.02267-19 PMC6775464 31575776

[B51] Lee J. Moir R. D. Mcintosh K. B. Willis I. M. (2012). TOR signaling regulates ribosome and tRNA synthesis via LAMMER/Clk and GSK-3 family kinases. Mol. Cell 45, 836–843. doi: 10.1016/j.molcel.2012.01.018 22364741 PMC3319249

[B52] Lee S.-B. Mota C. Thak E. J. Kim J. Son Y. J. Oh D.-B.. (2023). Effects of altered *N*-glycan structures of *Cryptococcus neoformans* mannoproteins, MP98 (Cda2) and MP84 (Cda3), on interaction with host cells. Sci. Rep. 13, 1175. doi: 10.1038/s41598-023-27422-9 36670130 PMC9859814

[B53] Li S. Zhao Y. Zhang Y. Zhang Y. Zhang Z. Tang C.. (2021). The Δ subunit of F1fo-atp synthase is required for pathogenicity of *Candida albicans* . Nat. Commun. 12, 6041. doi: 10.1038/s41467-021-26313-9 34654833 PMC8519961

[B54] Li L. Zhu X.-M. Wu J.-Q. Cao N. Bao J.-D. Liu X.-H.. (2022). The lammer kinase MoKns1 regulates growth, conidiation and pathogenicity in *Magnaporthe oryzae* . Int. J. Mol. Sci. 23, 8104. doi: 10.3390/ijms23158104 35897680 PMC9332457

[B55] Lim J.-Y. Kim Y. J. Woo S. A. Jeong J. W. Lee Y.-R. Kim C.-H.. (2021). The lammer kinase, LkhA, affects *Aspergillus fumigatus* pathogenicity by modulating reproduction and biosynthesis of cell wall pamps. Front. Cell. Infect. Microbiol. 1001. doi: 10.3389/fcimb.2021.756206 PMC8548842 34722342

[B56] Lim J.-Y. Park Y.-H. Pyon Y.-H. Yang J.-M. Yoon J.-Y. Park S. J.. (2020). The lammer kinase is involved in morphogenesis and response to cell wall-and DNA-damaging stresses in *Candida albicans* . Med. Mycol. 58, 240–247. doi: 10.1093/mmy/myz049 31100152

[B57] Littman M. (1958). Capsule synthesis by *Cryptococcus neoformans* . Trans. Ny Acad. Sci. 20, 623–648. doi: 10.1111/j.2164-0947.1958.tb00625.x 13556861

[B58] Livak K. J. Schmittgen T. D. (2001). Analysis of relative gene expression data using real-time quantitative pcr and the 2^– Δδct^ method. Methods 25, 402–408. doi: 10.1006/meth.2001.1262 11846609

[B59] Manning G. Plowman G. D. Hunter T. Sudarsanam S. (2002). Evolution of protein kinase signaling from yeast to man. Trends. Biochem. Sci. 27, 514–520. doi: 10.1016/S0968-0004(02)02179-5 12368087

[B60] Martho K. F. De Melo A. T. Takahashi J. P. Guerra J. M. Santos D. C. Purisco S. U.. (2016). Amino acid permeases and virulence in *Cryptococcus neoformans* . PloS One 11, E0163919. doi: 10.1371/journal.pone.0163919 27695080 PMC5047642

[B61] Miller R. A. Britigan B. E. (1997). Role of oxidants in microbial pathophysiology. Clin. Microbiol. Rev. 10, 1–18. doi: 10.1128/CMR.10.1.1 8993856 PMC172912

[B62] Myers M. P. Murphy M. B. Landreth G. (1994). The dual-specificity Clk kinase induces neuronal differentiation of PC12 cells. Mol. Cell. Biol. 14, 6954–6961. doi: 10.1128/mcb.14.10.6954-6961.1994 7935412 PMC359226

[B63] Padmanabha R. Gehrung S. Snyder M. (1991). The *kns1* gene of *Saccharomyces cerevisiae* encodes A nonessential protein kinase homologue that is distantly related to members of the *cdc28*/*cdc2* gene family. Mol. Gen. Genet. 229, 1–9. doi: 10.1007/BF00264206 1910150

[B64] Park Y.-D. Kang W.-H. Yang W.-S. Shin K.-S. Bae K. S. Park H.-M. (2003). Lammer kinase homolog, Lkh1, is involved in oxidative-stress response of fission yeast. Biochem. Biophys. Res. Commun. 311, 1078–1083. doi: 10.1016/j.bbrc.2003.10.110 14623292

[B65] Park Y.-H. Park H.-M. (2011). Disruption of the dual specificity kinase gene causes the reduction of virulence in *Candida albicans* . Kor. J. Mycol. 39, 85–87. doi: 10.4489/KJM.2011.39.1.085

[B66] Park Y.-H. Yang J.-M. Yang S.-Y. Kim S.-M. Cho Y.-M. Park H.-M. (2011). Function of dual specificity kinase, ScKns1, in adhesive and filamentous growth of *Saccharomyces cerevisiae* . Korean J. Microbiol. 47, 110–116.

[B67] Pedruzzi I. Dubouloz F. Cameroni E. Wanke V. Roosen J. Winderickx J.. (2003). TOR and PKA signaling pathways converge on the protein kinase Rim15 to control entry into G0. Mol. Cell. Biol. 12, 1607–1613. doi: 10.1016/S1097-2765(03)00485-4 14690612

[B68] Perfect J. R. (2014). Cryptococcosis: A model for the understanding of infectious diseases. J. Clin. Invest. 124, 1893–1895. doi: 10.1172/JCI75241 24743152 PMC4001560

[B69] Price M. S. Betancourt-Quiroz M. Price J. L. Toffaletti D. L. Vora H. Hu G.. (2011). *Cryptococcus neoformans* requires A functional glycolytic pathway for disease but not persistence in the host. Mbio 2, E00103–E00111. doi: 10.1128/mBio.00103-11 21652778 PMC3110414

[B70] Rajasingham R. Govender N. P. Jordan A. Loyse A. Shroufi A. Denning D. W.. (2022). The global burden of hiv-associated cryptococcal infection in adults in 2020: A modelling analysis. Lancet Infect. Dis. 22, 1748–1755. doi: 10.1016/S1473-3099(22)00499-6 36049486 PMC9701154

[B71] Reese A. J. Doering T. L. (2003). Cell wall α-1, 3-glucan is required to anchor the *Cryptococcus neoformans* capsule. Mol. Microbiol. 50, 1401–1409. doi: 10.1046/j.1365-2958.2003.03780.x 14622425

[B72] Reese A. J. Yoneda A. Breger J. A. Beauvais A. Liu H. Griffith C. L.. (2007). Loss of cell wall alpha (1-3) glucan affects *Cryptococcus neoformans* from ultrastructure to virulence. Mol. Microbiol. 63, 1385–1398. doi: 10.1111/j.1365-2958.2006.05551.x 17244196 PMC1864955

[B73] Regalla D. Vannatta M. Alam M. Malek A. E. (2022). Covid-19-associated *Cryptococcus* infection (Caci): A review of literature and clinical pearls. Infection 50, 1007–1012. doi: 10.1007/s15010-022-01805-y 35322336 PMC8942802

[B74] Ristow L. C. Jezewski A. J. Chadwick B. J. Stamnes M. A. Lin X. Krysan D. J. (2023). *Cryptococcus neoformans* adapts to the host environment through TOR-mediated remodeling of phospholipid asymmetry. Nat. Commun. 14, 6587. doi: 10.1038/s41467-023-42318-y 37852972 PMC10584969

[B75] Sanchez-Casalongue M. E. Lee J. Diamond A. Shuldiner S. Moir R. D. Willis I. M. (2015). Differential phosphorylation of A regulatory subunit of protein kinase CK2 by target of rapamycin complex 1 signaling and the Cdc-like kinase Kns1. J. Biol. Chem. 290, 7221–7233. doi: 10.1074/jbc.M114.626523 25631054 PMC4358141

[B76] Sanguinetti M. Posteraro B. La Sorda M. Torelli R. Fiori B. Santangelo R.. (2006). Role of *afr1*, an abc transporter-encoding gene, in the *in vivo* response to fluconazole and virulence of *Cryptococcus neoformans* . Infect. Immun. 74, 1352–1359. doi: 10.1128/IAI.74.2.1352-1359.2006 16428784 PMC1360305

[B77] Santiago-Tirado F. H. Onken M. D. Cooper J. A. Klein R. S. Doering T. L. (2017). Trojan horse transit contributes to blood-brain barrier crossing of A eukaryotic pathogen. Mbio 8, E02183–E02116. doi: 10.1128/mBio.02183-16 28143979 PMC5285505

[B78] Savaldi-Goldstein S. Aviv D. Davydov O. Fluhr R. (2003). Alternative splicing modulation by A lammer kinase impinges on developmental and transcriptome expression. Plant Cell 15, 926–938. doi: 10.1105/tpc.011056 12671088 PMC152339

[B79] Schmelzle T. Beck T. Martin D. E. Hall M. N. (2004). Activation of the RAS/cyclic AMP pathway suppresses A TOR deficiency in yeast. Mol. Cell. Biol. 24, 338–351. doi: 10.1128/MCB.24.1.338-351.2004 PMC303340 14673167

[B80] Shahi G. Kumar M. Skwarecki A. S. Edmondson M. Banerjee A. Usher J.. (2022). Fluconazole resistant *Candida auris* clinical isolates have increased levels of cell wall chitin and increased susceptibility to A glucosamine-6-phosphate synthase inhibitor. Cell. Surf. 8, 100076. doi: 10.1016/j.tcsw.2022.100076 35252632 PMC8891998

[B81] So Y.-S. Lee D.-G. Idnurm A. Ianiri G. Bahn Y.-S. (2019). The TOR pathway plays pleiotropic roles in growth and stress responses of the fungal pathogen *Cryptococcus neoformans* . Genetics 212, 1241–1258. doi: 10.1534/genetics.119.302191 31175227 PMC6707454

[B82] So Y.-S. Yang D.-H. Jung K.-W. Huh W.-K. Bahn Y.-S. (2017). Molecular characterization of adenylyl cyclase complex proteins using versatile protein-tagging plasmid systems in *Cryptococcus neoformans* . J. Microbiol. Biotechnol. 27, 357–364. doi: 10.4014/jmb.1609.09036 27780958

[B83] Song M. Pang L. Zhang M. Qu Y. Laster K. V. Dong Z. (2023). Cdc2-like kinases: structure, biological function, and therapeutic targets for diseases. Signal. Transduction Targeting Ther. 8, 148. doi: 10.1038/s41392-023-01409-4 PMC10082069 37029108

[B84] Takayama S. Fujii M. Kurosawa A. Adachi N. Ayusawa D. (2007). Overexpression of *HAM1* gene detoxifies 5-bromodeoxyuridine in the yeast *Saccharomyces cerevisiae* . Curr. Genet. 52, 203–211. doi: 10.1007/s00294-007-0152-z 17899088

[B85] Talmadge C. B. Finkernagel S. Sumegi J. Sciorra L. Rabinow L. (1998). Chromosomal mapping of three human lammer protein-kinase-encoding genes. Hum. Genet. 103, 523–524. doi: 10.1007/s004390050861 9856501

[B86] Teixeira P. A. Penha L. L. Mendonéa-Previato L. Previato J. O. (2014). Mannoprotein mp84 mediates the adhesion of *Cryptococcus neoformans* to epithelial lung cells. Front. Cell. Infect. Microbiol. 4, 106. doi: 10.3389/fcimb.2014.00106 25191644 PMC4137752

[B87] Thompson J. R. Douglas C. M. Li W. Jue C. K. Pramanik B. Yuan X.. (1999). A glucan synthase fks1 homolog in *Cryptococcus neoformans* is single copy and encodes an essential function. J. Bacteriol. 181, 444–453. doi: 10.1128/JB.181.2.444-453.1999 9882657 PMC93397

[B88] Urban J. Soulard A. Huber A. Lippman S. Mukhopadhyay D. Deloche O.. (2007). Sch9 is A major target of TORC1 in *Saccharomyces cerevisiae* . Mol. Cell 26, 663–674. doi: 10.1016/j.molcel.2007.04.020 17560372

[B89] Veen M. Stahl U. Lang C. (2003). Combined overexpression of genes of the ergosterol biosynthetic pathway leads to accumulation of sterols in *Saccharomyces cerevisiae* . FEMS Yeast Res. 4, 87–95. doi: 10.1016/S1567-1356(03)00126-0 14554200

[B90] Velagapudi R. Hsueh Y. P. Geunes-Boyer S. Wright J. R. Heitman J. (2009). Spores as infectious propagules of *Cryptococcus neoformans* . Infect. Immun. 77, 4345–4355. doi: 10.1128/IAI.00542-09 19620339 PMC2747963

[B91] Vestergaard M. Bald D. Ingmer H. (2022). Targeting the atp synthase in bacterial and fungal pathogens: beyond *Mycobacterium tuberculosis* . J. Glob. Antimicrob. Resist. 29, 29–41. doi: 10.1016/j.jgar.2022.01.026 35131507

[B92] Vu K. Weksler B. Romero I. Couraud P.-O. Gelli A. (2009). Immortalized human brain endothelial cell line hcmec/D3 as A model of the blood-brain barrier facilitates *in vitro* studies of central nervous system infection by *Cryptococcus neoformans* . Eukaryot. Cell. 8, 1803–1807. doi: 10.1128/EC.00240-09 19767445 PMC2772405

[B93] Wang Z. A. Li L. X. Doering T. L. (2018). Unraveling synthesis of the cryptococcal cell wall and capsule. Glycobiology 28, 719–730. doi: 10.1093/glycob/cwy030 29648596 PMC6142866

[B94] Yang M. Jarrett S. G. Craven R. Kaetzel D. M. (2009). Ynk1, the yeast homolog of human metastasis suppressor NM23, is required for repair of UV radiation-and etoposide-induced DNA damage. Mutat. Res. 660, 74–78. doi: 10.1016/j.mrfmmm.2008.09.015 18983998 PMC2746497

[B95] Young L. Y. Hull C. M. Heitman J. (2003). Disruption of ergosterol biosynthesis confers resistance to amphotericin B in *Candida lusitaniae* . Antimicrob. Agents. Chemother. 47, 2717–2724. doi: 10.1128/AAC.47.9.2717-2724.2003 12936965 PMC182600

[B96] Yu E.-Y. Lee J.-H. Kang W.-H. Park Y.-H. Kim L. Park H.-M. (2013). Fission yeast lammer kinase Lkh1 regulates the cell cycle by phosphorylating the Cdk-inhibitor Rum1. Biochem. Biophys. Res. Commun. 432, 80–85. doi: 10.1016/j.bbrc.2013.01.082 23376070

[B97] Yun B. Farkas R. Lee K. Rabinow L. (1994). The DOA locus encodes A member of A new protein kinase family and is essential for eye and embryonic development in. Drosophila Melanogaster. Genes Dev. 8, 1160–1173. doi: 10.1101/gad.8.10.1160 7926721

[B98] Zaragoza O. Rodrigues M. L. De Jesus M. Frases S. Dadachova E. Casadevall A. (2009). The capsule of the fungal pathogen *Cryptococcus neoformans* . Adv. Appl. Microbiol. 68, 133–216. doi: 10.1016/S0065-2164(09)01204-0 19426855 PMC2739887

