# Supplementary figures and images for: Pleiotropic roles of LAMMER kinase, Lkh1 in stress responses and virulence of Cryptococcus neoformans
Source: Front Cell Infect Microbiol. 2024 May 7;14:1369301. doi: 10.3389/fcimb.2024.1369301 (PMC11106425; doi:10.3389/fcimb.2024.1369301)

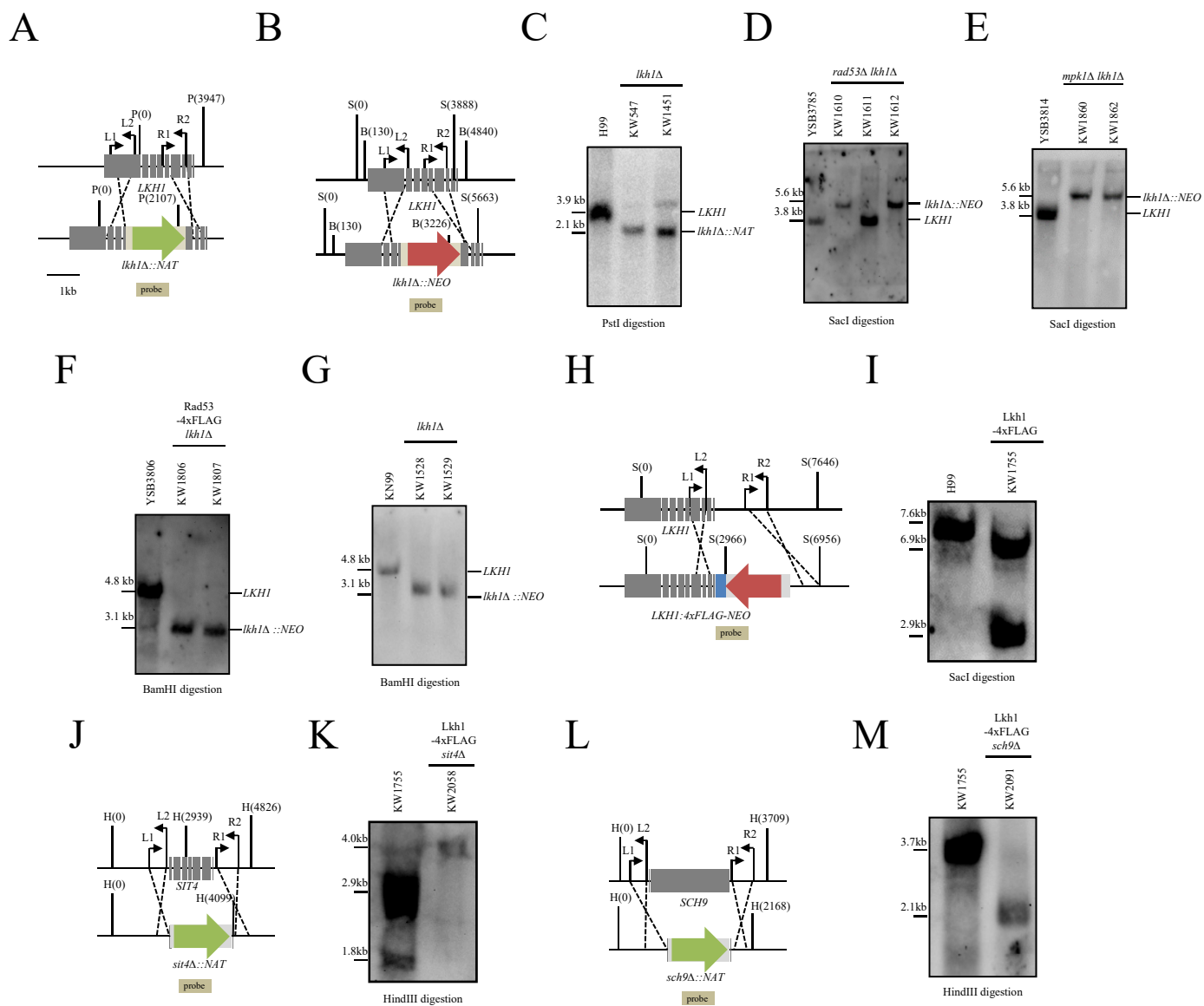

**Figure S1 (Kwon et al)**

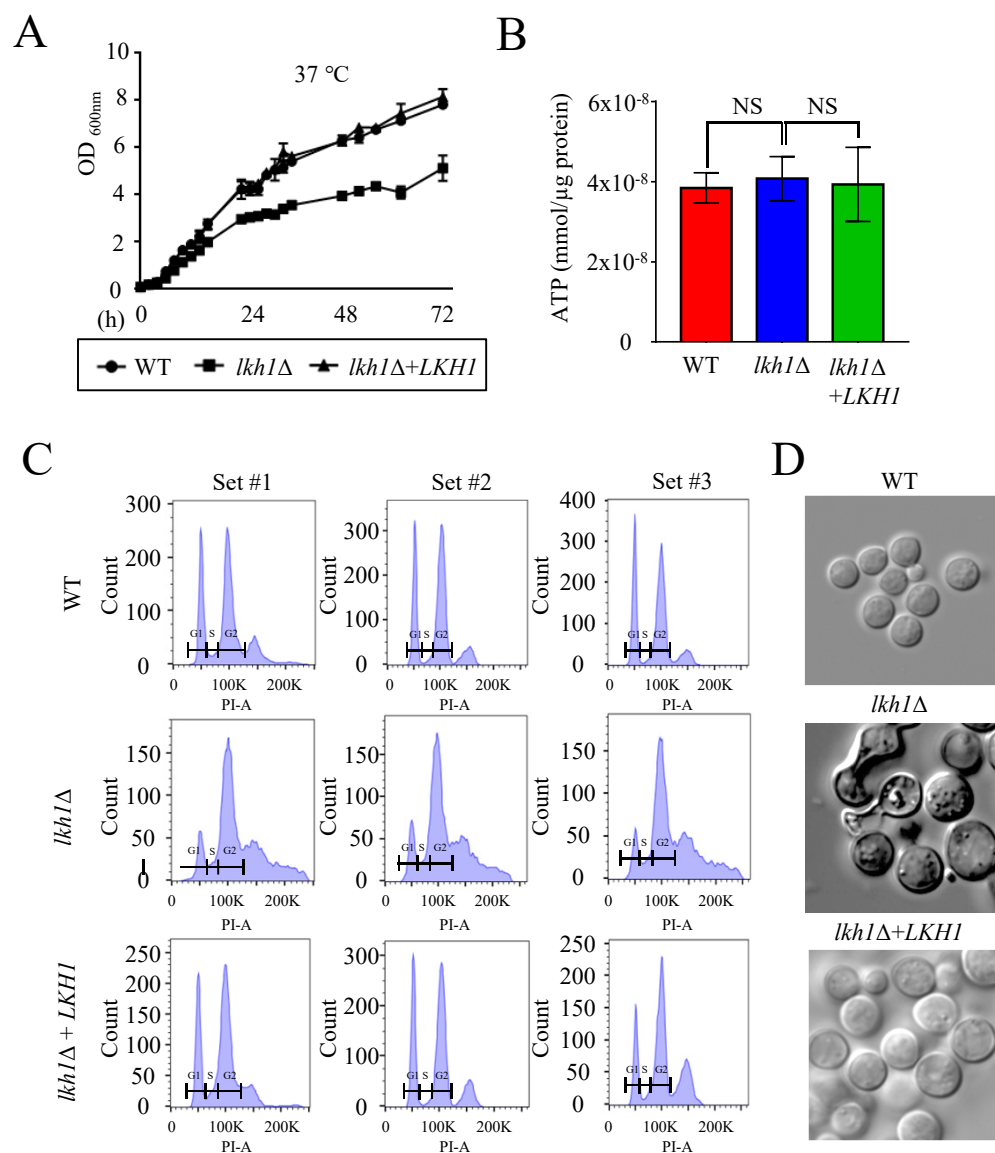

**Figure S2**  
(Kwon et al)

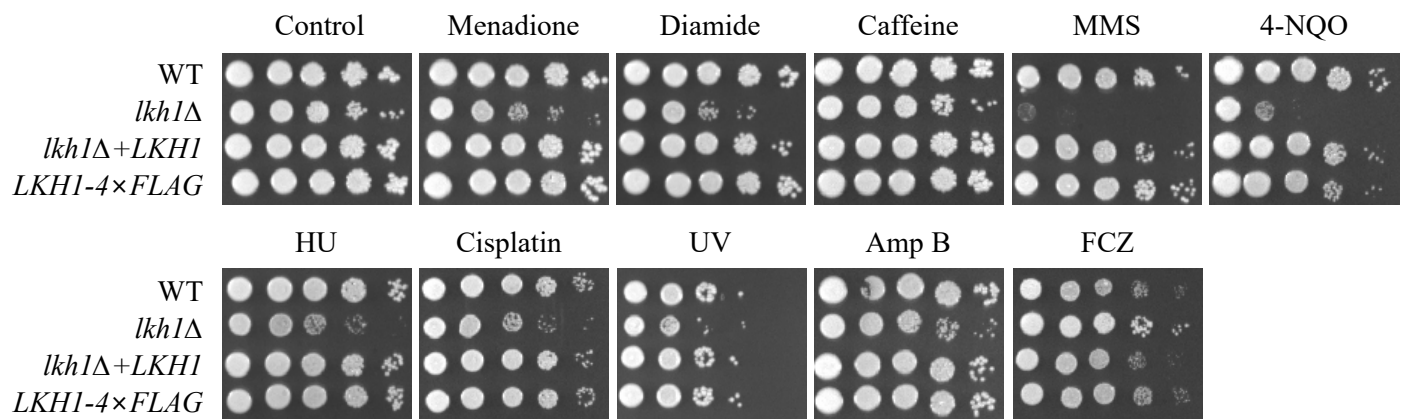

**Figure S3**  
(Kwon et al)

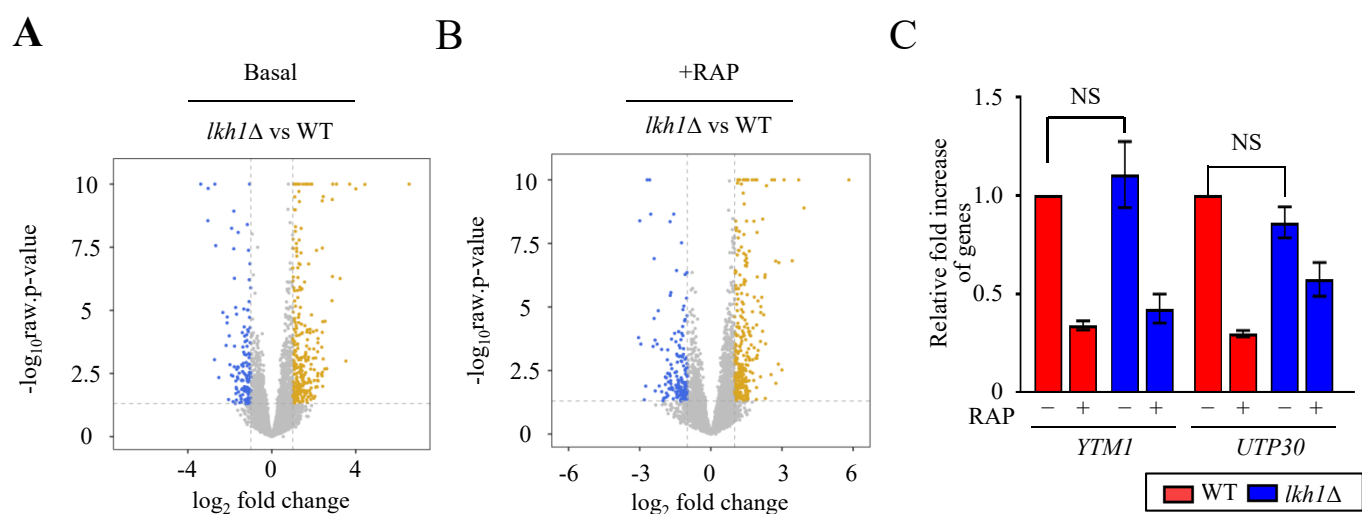

**Figure S4**  
(Kwon et al)

**A**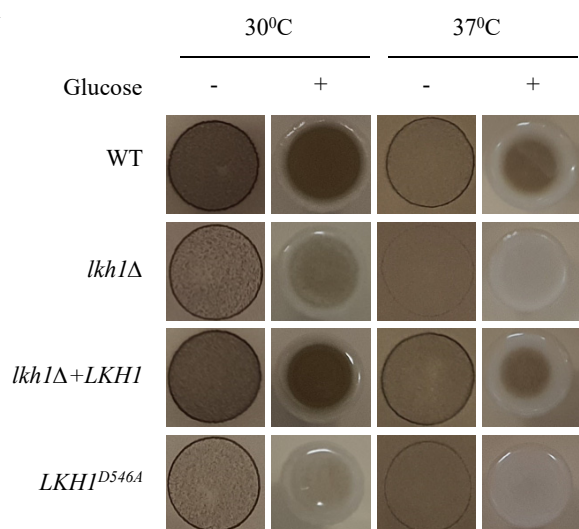**B**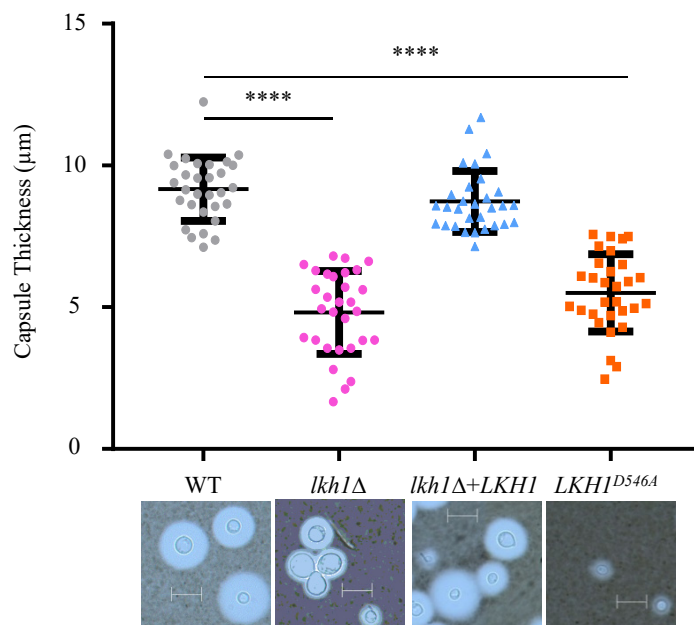

**Figure S5**  
(Kwon et al)

Supplement: Supplementary Figure 1 — Construction of the lkh1Δ, rad53Δ lkh1Δ, mpk1Δ lkh1Δ, Rad53-4×FLAG lkh1Δ, LKH1-4×FLAG, LKH1-4×FLAG sit4Δ, and LKH1-4×FLAG sch9Δ mutants. (A, B, H, J, L) Diagram for LKH1 gene disruption in serotype A (MATα) H99 strain, rad53Δ, mpk1Δ, Rad53-4×FLAG mutant, MATa KN99, LKH1-4×FLAG, LKH1-4×FLAG sit4Δ, and LKH1-4×FLAG sch9Δ mutants. (C, D, E, F, G, I, K, and M) The correct gene disruptions were confirmed by Southern blot analysis using genomic DNAs digested with the indicated restriction enzyme. [file DataSheet_1.pdf]
