# Supplementary material for: Pleiotropic roles of LAMMER kinase, Lkh1 in stress responses and virulence of Cryptococcus neoformans
Source: Front Cell Infect Microbiol. 2024 May 7;14:1369301. doi: 10.3389/fcimb.2024.1369301 (PMC11106425; doi:10.3389/fcimb.2024.1369301)
Supplement: Supplementary file 2 [file Table_1.docx]

Table S1. Strains used in this study

| Strain | Genotype | Parent | Reference |
| --- | --- | --- | --- |
| H99 | *MAT*α |  | (Perfect et al., 1993) |
| YSB3785 | *MAT*α *CNAG_05216* (*RAD53*)*::NAT* | H99 | (Jung et al., 2019) |
| KN99 | *MAT***a** |  | (Nielsen et al., 2003) |
| YSB3814 | *MAT*α *CNAG_04514* (*MPK1*)*::NAT* | H99 | (Lee et al., 2016) |
| YSB3806 | *MAT*α *CNAG_05216-4*×*FLAG-NEO* | H99 | (Jung et al., 2019) |
| YSB4095 | *MAT*α *CNAG_01436* (*SIT4*)*::NAT* | H99 | (Jin et al., 2020) |
| KW1451 | *MAT*α *CNAG_00683* (*LKH1*)*::NAT#43* | H99 | This study |
| KW1610 | *MAT*α *CNAG_05216::NAT CNAG_00683::NEO* | YSB3785 | This study |
| KW1612 | *MAT*α *CNAG_05216::NAT CNAG_00683::NEO* | YSB3785 | This study |
| KW1528 | *MAT***a** *CNAG_00683::NEO* | KN99 | This study |
| KW1529 | *MAT***a** *CNAG_00683::NEO* | KN99 | This study |
| KW1633 | *MAT*α *CNAG_00683::NAT pNEO-CNAG_00683*) | KW1451 | This study |
| KW1860 | *MAT*α *CNAG_04514::NAT CNAG_00683*)*::NEO* | YSB3814 | This study |
| KW1862 | *MAT*α *CNAG_04514::NAT CNAG_00683::NEO* | YSB3814 | This study |
| KW1806 | *MAT*α *CNAG_05216-4*×*FLAG-NEO CNAG_00683::NEO* | YSB3806 | This study |
| KW1807 | *MAT*α *CNAG_05216-4*×*FLAG-NEO CNAG_0068::NEO* | YSB3806 | This study |
| KW1755 | *MAT*α *CNAG_00683-4*×*FLAG-NEO* | H99 | This study |
| KW1958 | *MAT*α *CNAG_00683::NAT pNEO CNAG_00683 D546A* | KW1451 | This study |
| KW2058 | *MAT*α *CNAG_00683-4*×*FLAG-NEO CNAG_01436::NAT* | KW1755 | This study |
| KW2091 | *MATα CNAG_00683-4×FLAG-NEO CNAG_06301 (SCH9)::NAT* | KW1755 | This study |

# Reference

Jin, J.-H., Lee, K.-T., Hong, J., Lee, D., Jang, E.-H., Kim, J.-Y., Lee, Y., LeE, S.-H., So, Y.-S. & Jung, K.-W. 2020. Genome-wide functional analysis of phosphatases in the pathogenic fungus *Cryptococcus neoformans*. *Nat. Commun.,* 11**,** 4212. doi: 10.1038/s41467-020-18028-0

Jung, K.-W., Lee, Y., Huh, E. Y., Lee, S. C., Lim, S. & Bahn, Y.-S. 2019. Rad53-and Chk1-dependent DNA damage response pathways cooperatively promote fungal pathogenesis and modulate antifungal drug susceptibility. *MBio,* 10**,** e01726-18. doi: 10.1128/mBio.01726-18

Lee, K.-T., So, Y.-S., Yang, D.-H., Jung, K.-W., Choi, J., Lee, D.-G., Kwon, H., Jang, J., Wang, L. L. & Cha, S. 2016. Systematic functional analysis of kinases in the fungal pathogen *Cryptococcus neoformans*. *Nat. Commun.,* 7**,** 1-16. doi: 10.1038/ncomms12766

Nielsen, K., Cox, G. M., Wang, P., Toffaletti, D. L., Perfect, J. R. & Heitman, J. 2003. Sexual cycle of *Cryptococcus neoformans var. grubi*i and virulence of congenic a and α isolates. *Infect. Immun.,* 71**,** 4831-4841. doi: 10.1128/IAI.71.9.4831-4841.2003

Perfect, J., Ketabchi, N., Cox, G., Ingram, C. & Beiser, C. 1993. Karyotyping of *Cryptococcus neoformans* as an epidemiological tool. *J. Clin. Microbiol.,* 31**,** 3305-3309. doi: 10.1128/jcm.31.12.3305-3309.1993
