# Supplementary material for: Pleiotropic roles of LAMMER kinase, Lkh1 in stress responses and virulence of Cryptococcus neoformans
Source: Front Cell Infect Microbiol. 2024 May 7;14:1369301. doi: 10.3389/fcimb.2024.1369301 (PMC11106425; doi:10.3389/fcimb.2024.1369301)
Supplement: Supplementary file 3 [file Table_2.docx]

## Table S2. Primers used in this study

| Primer Name | Sequence (5’-3’) | Comment |
| --- | --- | --- |
| B79 | TGTGGATGCTGGCGGAGGATA | Screening primer on *ACT1* promoter |
| B1026 | GTAAAACGACGGCCAGTGAGC | M13 forward (extended) |
| B1027 | CAGGAAACAGCTATGACCATG | M13 reverse (extended) |
| B1454 | AAGGTGTTCCCCGACGACGAATCG | NSL-NAT |
| B1455 | AACTCCGTCGCGAGCCCCATCAAC | NSR-NAT |
| B1886 | TGGAAGAGATGGATGTGC | NSL-NEO |
| B1887 | ATTGTCTGTTGTGCCCAG | NSR-NEO |
| B6567 | GCATGCGGCGCGCCAGAT | 4×FLAG NEO marker*-* left flanking primer 1 |
| B354 | GCATGCAGGATTCGAGTG | 4×FLAG NEO marker*-* right flanking primer 1 |
| J787 | GGACAACTGAGCACACCTAC | *LKH1-*left flanking primer 1 |
| J788 | TCACTGGCCGTCGTTTTACACTCATCCCGTACAACTCG | *LKH1*-left flanking primer 2 |
| J326 | GTCATAGCTGTTTCCTGGGTTCTGCCACTTTTGAATC | *LKH1*-right flanking primer 1 |
| J790 | AGAAAAACTCGGACACCTG | *LKH1*-right flanking primer 2 |
| J791 | TAAGGCTGCCGATCTCTTG | *LKH1*-screening primer |
| J718 | GCGGCCGCTTTTGTGCTGCGACCGTAAGCC | *LKH1*-L primer 1 for complementation |
| J723 | TTGGACGGTTCGGCCTTTC | *LKH1*-L primer 2 for complementation |
| J1711 | CGCGGATCCCTCCGCCGCTGAC | *LKH1*-R primer 1 for complementation |
| J1712 | CGCGGATCCGCGGCCGCTCACAGTGTACGTCCGTTA | *LKH1*-R primer 2 for complementation |
| J1893 | GCGGCCGCCAGATCTTCCGGATGGCGCATGCAGGATTCGAGTG | *LKH1*-LR primer 1 for complementation |
| J1894 | AAAGCGGCCGCTCTTGCTGAAAAACGGAGCCATGAAGATCCTGA | *LKH1*-LR primer 2 for complementation |
| J719 | GATGTGTCTATGAGGCACG | *LKH1*-primer 2 for sequencing |
| J786 | AAGAACTCGCAGAGGGCGCA | *LKH1*-primer 3 for sequencing |
| J1120 | CACTGTGGCTCCGTCCAATG | *LKH1*-primer 4 for sequencing |
| J1813 | CTCGCCTCGTTTGAACATAGA | *LKH1*-probe primer for southern blot |
| J1966 | TCAGGATCTTCATGGCTCCATTCGCGTTCCATGTCCTCC | *LKH1*-left flanking primer 1 for FLAG tagging |
| J1967 | ATCTGGCGCGCCGCATGCTGCGGGCTCTGGAATAGGTTCG | *LKH1*-left flanking primer 2 for FLAG tagging |
| J1968 | CCACTCGAATCCTGCATGCTAGTTGGAACTTGTCATAGAAT | *LKH1*-right flanking primer 1 for FLAG tagging |
| J1969 | TTCCCACAACATGCTTGC | *LKH1*-right flanking primer 2 for FLAG tagging |
| J1970 | CCCTTCTCAATGGGTTCCTG | *LKH1*-4xFLAG screening primer |
| J2006 | TCAAGCTCGTACACACCGCCCTCAAGCCGGAGAATAT | *LKH1* for point mutation for Aspartic acid (alanine) primer 1 |
| J2007 | ATATTCTCCGGCTTGAGGGCGGTGTGTACGAGCTTGA | *LKH1* for point mutation for Aspartic acid (alanine) primer 2 |
| J1025 | TTTGCATTAGAACTAAAAACAAAGCA | CnU6 promoter primer 1 for SgRNA cassette |
| J1026 | AAAAAAGCACCGACTCGGT | gRNA design primer 1 for SgRNA cassette |
| J1147 | TTTTGACGGGATAAAAACACAACAGTATACCCTGCCGGTG | *LKH1*- U6 promoter primer 2 for SgRNA cassete |
| J1148 | GTGTTTTTATCCCGTCAAAAGTTTTAGAGCTAGAAATAGCAAGTT | *LKH1-* gRNA design primer 2 for SgRNA cassette |
| J1023 | GGTGACGCTGTGAGAGTGG | CAS9 promoter primer 1 |
| J1024 | GGGCCCCTCTTCACGTGG | CAS9 promoter primer 2 |
| B7242 | AGTGAGGTAGAAACCACGG | *SIT4-*left flanking primer 1 |
| B7243 | TCACTGGCCGTCGTTTTACTGAAGAGATTGGGATGGG | *SIT4-*left flanking primer 2 |
| B7244 | CATGGTCATAGCTGTTTCCTGACTAATCTGTCCTGGCTGG | *SIT4-*right flanking primer 1 |
| B7245 | CAAGGGTCTAAAGGAAGTCC | *SIT4-*right flanking primer 2 |
| B7246 | GATACCCCAAGTGTCCCTAC | *SIT4* screening primer |
| B7247 | CATTACCGCATCTGTAGCAG | *SIT4* probe primer for southern blot |
| J399 | ACCAACGGCTGTACCTCTG | *SOD1* qRT primer 1 |
| J400 | ATATTACCGAGGTCACCAACG | *SOD1* qRT primer 2 |
| J401 | CAAGGCCACCAAGAAGCTC | *SOD2* qRT primer 1 |
| J402 | TAGAAAGCGTGCTCCCAG | *SOD2* qRT primer 2 |
| J403 | TTATTCACGCGGTAAGGGAG | *CAT1* qRT primer 1 |
| J404 | GAAATCTCCAGGCAAGCAAG | *CAT1* qRT primer 2 |
| J405 | ACCGAGACAACGGATGAGG | *CAT2* qRT primer 1 |
| J406 | TGACACGAGACATTGGTGATC | *CAT2* qRT primer 2 |
| J407 | TGCCTTCGTATCCAAGCTC | *CAT3* qRT primer 1 |
| J408 | ATTGCGTAGCATTGCCGAC | *CAT3* qRT primer 2 |
| J409 | ATCGCCTGATCTTGGAGC | *CAT4* qRT primer 1 |
| J410 | CGCCAAAGCCATTATGAGTAC | *CAT4* qRT primer 2 |
| J1 | TGGTGGTGAGGGAAAATG | *RAD51* qRT primer 1 |
| J2 | GCACCTCTTCACCATCAAG | *RAD51* qRT primer 2 |
| J3 | AAAGCGATGAGGATGACC | *RDH54* qRT primer 1 |
| J4 | CTCGTCTTTTTCAGCACG CACTTTCAGCCGATCCTAATTCAC | *RDH54* qRT primer 2 |
| J5 | GCAAAATCTTTCAGCGTGTG | *RAD54* qRT primer 1 |
| J6 | CACAAAGTTTCGGGGTTG | *RAD54* qRT primer 2 |
| J254 | ATATCAACCGCCGATCAGCAC | *BDR1* qRT primer 1 |
| J255 | TCCCTAATTCGTTGTTCACGCAC | *BDR1* qRT primer 2 |
| J2121 | GTTGTACTGTTGTTCCTTGGTTCG | *CHS2* qRT primer 1 |
| J2122 | TAAGGATTGAAAGTCGAGCTGGTC | *CHS2* qRT primer 2 |
| J2129 | CTGATATTCTCGTGGTTCGCTTTG | *CHS6* qRT primer 1 |
| J2130 | CCAAAGCACCAAGGTAACCATATC | *CHS6* qRT primer 2 |
| J2131 | GATGAGAAACCCAACCTTGACG | *CHS7* qRT primer 1 |
| J2132 | CATTGAGGGTTTGTATGGAGATGG | *CHS7* qRT primer 2 |
| J2133 | GAGCTGGTCTACCAAACATTGAAC | *CHS8* qRT primer 1 |
| J2134 | GAGCCGAGAGAGTTGAGAGATTGG | *CHS8* qRT primer 2 |
| J2135 | CACTTTCAGCCGATCCTAATTCAC | *KRE5* qRT primer 1 |
| J2136 | ACAACCAGTCTTGATCCAGTG | *KRE5* qRT primer 2 |
| J2137 | CAATGGGACAACACTTCAACAAGC | *KRE6* qRT primer 1 |
| J2138 | ACGGGTAAGAGAAGAGACAGATTG | *KRE6* qRT primer 2 |
| J2171 | CCACTTCCTTCCAGCTCAG | *AGS1* qRT primer 1 |
| J2172 | AAAGGGTGTACCATGCGAC | *AGS1* qRT primer 2 |
| J2173 | AGCTATTCTCCGACTGTGC | *FKS1* qRT primer 1 |
| J2174 | GGCACCACCGAAAGTCAAG | *FKS1* qRT primer 2 |
| J61 | TTCCCGCCTCACTTCAATC | *ERG1* qRT primer 1 |
| J62 | AGGAAGACCCTGGATGGAG | *ERG1* qRT primer 2 |
| J1340 | ACGGTGTTGAAGACGACAG | *HMG1* qRT primer 1 |
| J1341 | CCGCGTGAGCGTTAAATC | *HMG1* qRT primer 2 |
| B677 | AATCTCCTTACCAGCCATTCGG | *ERG11* qRT primer 1 |
| B678 | TTCAGGGAACTTGGGAACAGC | *ERG11* qRT primer 2 |
| J96 | TCTGGATACAGGGCGGAAG | *LAC1* qRT primer 1 |
| J97 | TCCCTCAGTAAGATGCCAC | *LAC1* qRT primer 2 |
| J98 | GTTTCAGTTGATGGGACGAGG | *LAC2* qRT primer 1 |
| J99 | TCCAAATCGTATCCTTCCTCG | *LAC2* qRT primer 2 |
| J2209 | CCCTCCTCAATCGTCCCTTGTC | *BRX1* qRT primer 1 |
| J2210 | CAGAGTTGGAGAAGATAGTGGGACC | *BRX1* qRT primer 2 |
| J2211 | CCGAAGAAGCCGAGATTTGGAAG | *MAK21* qRT primer 1 |
| J2212 | GTCACTGGAGCCGCTAATACTAATG | *MAK21* qRT primer 2 |
| J2213 | AGGAGAATTGGCAGGCAAGC | *RRP5* qRT primer 1 |
| J2214 | ACGGTCCCGAAGGCAATCTC | *RRP5* qRT primer 2 |
| J1988 | CTCGGCATAACGTGTTTGG | *NIP7* qRT primer 1 |
| J1989 | ATGTGCTTTGGCAACGTG | *NIP7* qRT primer 2 |
| J1990 | AAGACGAGCGGCAGTGTAATG | *UTP30* qRT primer 1 |
| J1991 | GGCGACGGAGGTTTCTGATTTC | *UTP30* qRT primer 2 |
| J1992 | TGCTGCTGGAGTGTTGAGG | *YTM1* qRT primer 1 |
| J1993 | TCGTCCTGTCCATGTTACC | *YTM1* qRT primer 2 |
| J2199 | TCTCAGTCGAGACGATTTCCCATAC | *AAP4* qRT primer 1 |
| J2200 | CCCTCCAAGAAGACAGTGAAACC | *AAP4* qRT primer 2 |
| J2195 | CTACCTCCAAGAACGCAAGAAATG | *PUT1* qRT primer 1 |
| J2196 | GTGGTGAGGATGGTAGAGATGGAG | *PUT1* qRT primer 2 |
| J2197 | GCTTTCGGATGTTTGGCCTATCTC | *AAP7* qRT primer 1 |
| J2198 | GTCCAGGTCACAAGATTGGCAG | *AAP7* qRT primer 2 |
| J2193 | TCAGTCGAGACGATTTCCCATACC | *AAP2* qRT primer 1 |
| J2194 | GCCCTCCAAGAAGACAGTGAAAC | *AAP2* qRT primer 2 |
| B679 | CGCCCTTGCTCCTTCTTCTATG | *ACT1* qRT primer 1 |
| B680 | GACTCGTCGTATTCGCTCTTCG | *ACT1* qRT primer 2 |
| J2228 | ATTCATTCCCGATTGGCG | *CAP10* qRT primer 1 |
| J2229 | CCTTCTTTGAGATTGGCACG | *CAP10* qRT primer 2 |
| J2230 | CAAATCTTCCACCACACTGAATCC | *CAP59* qRT primer 1 |
| J2231 | GAGTGGGATCGAGAACAGCAATAC | *CAP59* qRT primer 2 |
| J2232 | ACGCTATGAACGAAGAGGC | *CAP60* qRT primer 1 |
| J2233 | GGAGTGAAAACAGAGTTGGG | *CAP60* qRT primer 2 |
| J2234 | CAAGGAAAGGGCATTCAGAG | *CAP64* qRT primer 1 |
| J2235 | ACTGGGACGGTCAGAAAG | *CAP64* qRT primer 2 |
| J2260 | TTCTTCGTGCTGAGAGGAG | *SCH9* screening primer |
| J2261 | AACCGAAACCCTCAGAACC | *SCH9* probe primer |
| J2262 | CCTTCGTCGTTCTTGTGTC | *SCH9-*left flanking primer 1 |
| J2263 | ATTCACTGGCCGTCGTTTTACAGATGTGGCGTAGTCAGCAC | *SCH9*-left flanking primer 2 |
| J2264 | CATGGTCATAGCTGTTTCCTGAATGAGAATGCGGTGGAC | *SCH9*-right flanking primer 1 |
| J2265 | GGATGGATGGATGCTCAT | *SCH9*-right flanking primer 2 |
| J2384 | AACGCGAAATCCAAGAACTGAAGG | *AFR1* qRT primer1 |
| J2385 | CAGTCTTGAGTTGAAAGAGGAAGC | *AFR1* qRT primer2 |
| J2386 | GTTCTCCATGTGTCTCATCTTCTG | *AFR2* qRT primer1 |
| J2387 | TCGACAAGGTAGGTGAATGGTGAG | *AFR2* qRT primer2 |
| J2388 | CTTGTTGTCTTTGTCGCCTCTCTC | *AFR3* qRT primer1 |
| J2389 | GTTGAGGATGGTCTCTGGAATAAG | *AFR3* qRT primer2 |
| J2390 | CGATGCCAAATACTCCACTGCTAG | *MDR1* qRT primer1 |
| J2391 | GGACGAAGGTGAAGACATTTCCAG | *MDR1* qRT primer2 |
| J2398 | CTCTACTCCTGCCTCTTCCAACTG | *MP88* qRT primer1 |
| J2399 | CCGAGGCAGTAGAGTTGACAGAG | *MP88* qRT primer2 |
| J2400 | CACTGCATACAACATCACCCAACC | *MP98* qRT primer1 |
| J2401 | GAGTGGTAGAGCTGCTGATGTTTG | *MP98* qRT primer2 |
| J2402 | GAAGGAAGTATTTGGCTTGGTGAC | *MP84* qRT primer1 |
| J2403 | GACCAAAGTGTCGAAGTAAGCCTC | *MP84* qRT primer2 |
